# Change of the aromatic nature through face-to-face stacking

**DOI:** 10.1039/d5sc10179d

**Published:** 2026-03-27

**Authors:** Qian Wang, Rinat T. Nasibullin, Dage Sundholm

**Affiliations:** a Department of Chemistry, Faculty of Science, University of Helsinki P.O. Box 55, A. I. Virtasen aukio 1 FIN-00014 Helsinki Finland dage.sundholm@helsinki.fi

## Abstract

We have computationally studied the aromatic nature of molecules built from tetraoxa-isophlorin (TOI) and Ni(ii)-norcorrole (NiNc) moieties. Calculations of the magnetically induced current density (MICD) susceptibility and magnetically induced ring currents (MIRC) show that antiaromatic porphyrinoids form bound aromatic stacked dimers with a distance of about 3 Å between the monomers. We design molecules consisting of many antiaromatic rings because they bind stronger due to the aromatic stabilization and a formal double bond between the individual pairs of the molecular rings of the stacked dimers. Global conjugation and strong coupling between the antiaromatic moieties can be prevented by introducing linkers with formal single bonds between the antiaromatic rings. Cyclooctatetraene (COT) with two ethynyl groups in the *para* positions is a good linker because the COT and TOI rings contribute to the aromatic binding by becoming aromatic in the stacked dimer. The ethynyl spacers decrease the steric interaction between the TOI rings of the monomer. Calculations with periodic boundary conditions on a surface consisting of TOI rings with ethynyl substituted COT rings as linker molecules showed that the monomer consists of individual antiaromatic rings that form an aromatic stacked dimer structure. The infinite monomer surface has an indirect band gap, whereas the TOI rings of the corresponding dimer is aromatic with a direct band gap. The conjugated pathway of NiNc-based molecules was extended by connecting NiNc rings directly or by ethynyl linkers. NiNc rings connected in the C_β_–C_meso_–C_β_ positions are weakly aromatic, whereas those connected in the C_β_–C_β_ positions are antiaromatic forming an aromatic dimer. Connecting the NiNc rings with two ethynyl bridges in the C_β_ positions leads to locally antiaromatic NiNc and an aromatic stacked dimer. Calculations with periodic boundary conditions on a linear band of NiNc rings show that the band forms a stacked double-decker structure with an intermolecular distance of 2.69 Å.

## Abstract 2

Antiaromatic porphyrinoid rings form stacked aromatic dimers with a delocalized double bond between each pair of the porphyrinoid rings.

## Graphical abstract

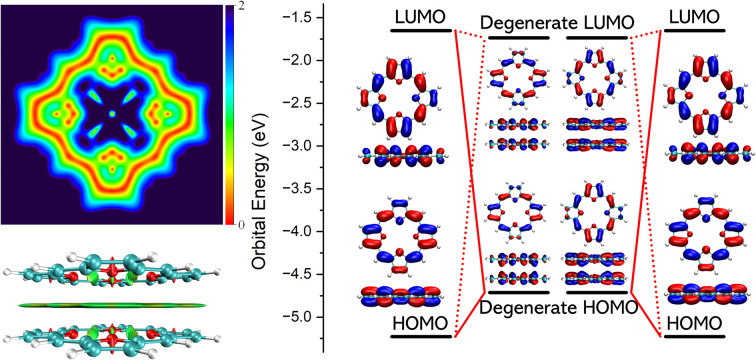

## 1 Introduction

Cram *et al.* studied in a long series of papers how strain and stacking affect molecular properties of benzene rings by synthesizing and characterizing molecules consisting of stacked aromatic rings.^1,2^ Corminboeuf, Schleyer and Warner extended the studies of stacked molecular rings by computationally investigating superphanes consisting of stacked antiaromatic rings and found that they are unexpectedly aromatic,^3^ which initiated a new research field.^4–15^ The change of the aromatic nature was explained by the reordering of energy levels of the frontier orbitals of cyclo-octatetraene (COT) when two planar COT molecules of *D*_8h_ symmetry form a stacked dimer.^3,5^ The highest-occupied molecular orbital (HOMO) of the COT molecule of *D*_8h_ symmetry is half filled. In the dimer, the binding combination of the HOMOs of the two COT molecules forms the doubly-degenerate closed-shell HOMO. The nature of the magnetically induced ring current (MIRC) of the antiaromatic monomer and the stacked aromatic rings were analyzed using an orbital model. The stacked COT dimer was found to be aromatic sustaining a net diatropic MIRC, whereas planar COT belonging to the *D*_4h_ symmetry is antiaromatic.^5^ Shinokubo *et al.* reported synthesis of molecules consisting of a Ni(ii)-norcorrole (NiNc) dimer that adopts a π-stacked structure with a short distance of about 3 Å between the two molecules.^16–21^ Nuclear magnetic resonance (NMR) spectroscopy and computational studies confirmed that the antiaromatic NiNc molecules become aromatic in the stacked dimer.^18,20,21^

The observed change in the aromatic nature upon dimerization can be understood using an extension of Hückel's aromaticity rule. Models like electrons on a circular disc^22^ and the perimeter model, which was introduced by Platt in 1949,^23,24^ can also be employed because they represent in the aromaticity context the same topology as electrons on a round ring. The angular wave function of electrons on a circular disc or on a round ring is exp(*imφ*), where *m* = 0, ±1, ±2, … is the quantum number of circular motion and *i* is the imaginary number.^22,25^ The Aufbau principle then leads to 2, 6, 10, … electrons in closed-shell systems corresponding to the number of electrons of a system with occupied σ, π, δ, … orbitals. This is an alternative view of the 4*n* + 2 electron Hückel rule for aromatic molecular rings and for antiaromatic ones having 4*n* electrons.^26,27^ A more general orbital counting rule states that molecules with odd number of occupied conjugated orbitals in the ring are aromatic and those having an even number of occupied conjugated orbitals are antiaromatic.^28^ The orbital-counting rule can be applied to closed-shell and open-shell molecules. Circular closed-shell molecular discs or round rings with many electrons but only two electrons in the highest-occupied molecular orbital (HOMO) are then antiaromatic. Adding or removing two electrons leads to aromaticity.

When two antiaromatic molecules approach each other from far away, each of them initially has two extra electrons, or they lack two electrons in the outermost shell. At short intermolecular distances, they can share these electrons leading to a delocalized double bond. The dimer has a reflection plane between the two molecules in addition to the angular dependence of the orbitals. The double ring of the dimer can be considered to have cylindrical topology as the B_20_ ring.^25,29,30^ In the dimer, the Aufbau principle leads to occupied σ_g_, σ_u_, π_u_, π_g_, δ_g_, δ_u_, … orbitals accumulating 2, 4, 8, 12, … electrons in the shells implying that 4*n* electrons close the shell for many-electron systems. At a short distance between the molecules, the binding combination of the HOMO of the two molecules forms a binding orbital (HOMO-1). The binding combination of the lowest occupied molecular orbitals (LUMO) is stabilized and becomes the binding HOMO of the dimer.^20,21^ The antibonding combinations of the HOMO and LUMO of the monomers become the LUMO and LUMO+1 of the dimer.

The non-interacting monomers are antiaromatic because the HOMO–LUMO transition is electric dipole forbidden but magnetic dipole allowed.^31^ Spectroscopically, the electronic dipole transition from HOMO to LUMO of the monomer is symmetry forbidden because the HOMO and LUMO have even parity and the parity of the dipole operator is odd. The transition is on the other hand magnetic dipole allowed leading to antiaromaticity.^32,33^ The HOMO–LUMO transition of the dimer is electric dipole allowed because the HOMO has even parity and the parity of the LUMO is odd. The formation of the dimer changes the aromatic nature from antiaromatic to aromatic.^21^

The binding mechanism can be applied to NiNc, whose dimer consists of two strongly interacting NiNc molecules in a face-to-face orientation.^16–21^ The Aufbau principle leads to closed shells and aromaticity for the dimer with 4*n* electrons in the conjugated orbitals. Similar changes in the aromatic nature are expected for dimers consisting of other strongly interacting antiaromatic rings such as tetraoxa-isophlorin (TOI), which we also study in this work. The ability to form a stable dimer depends on the radius of the conjugated ring and the degree of antiaromaticity of the monomer, which we determine by calculating the magnetically induced current density (MICD) susceptibility and the magnetically induced ring current (MIRC) strength using our gauge including magnetically induced currents (GIMIC) method.^25,34–37^ The bonding of the NiNc dimer is also strengthened by an attractive interaction between the Ni atoms.^20^

**Fig. 1:**
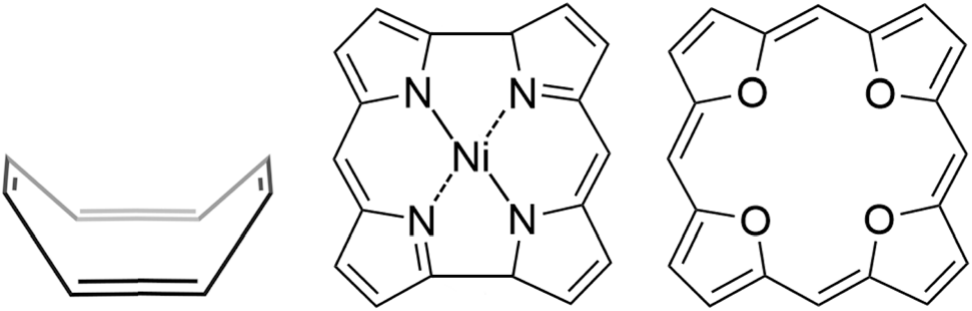
The molecular structure of COT, NiNc and TOI (from left to right).

In this work, we construct dimers of porphyrinoids and of linked porphyrinoids consisting of several independent antiaromatic rings or fused porphyrinoid rings to increase the bonding strength of the stacked dimer. The dimers are constructed by linking antiaromatic TOI molecules^38–46^ as well as by linking or fusing NiNc molecules, shown in Fig. 1. TOI and NiNc are strongly antiaromatic molecules with an even number of occupied conjugated orbitals in the molecular ring.^47^ The antiaromatic character can be adjusted by fusing the antiaromatic rings or by linking them *via* aromatic, antiaromatic or non-aromatic spacers.^48–52^

## 2 Computational methods

The molecular structures of tetraoxa-isophlorin (TOI) and its dimer were optimized at the density functional theory (DFT) level using the B3LYP,^53–55^ CAM-B3LYP,^56^ and ωB97X^57^ functionals and the def2-TZVP basis sets.^58^ Dispersion effects were considered using the empirical D3(BJ) term in the DFT calculations.^59^ Calculations of the vibrational frequencies showed that the optimized molecular structures are minima on the potential energy surface.^60^ The molecular structure of TOI and its dimer were also optimized at the second-order Møller–Plesset perturbation theory (MP2) level using def2-SVP basis set.^61,62^ The binding energies including corrections for basis-set superposition errors (BSSE)^63,64^ were obtained in single-point second-order Møller–Plesset perturbation theory (MP2) calculations using various basis sets.^58,61^ The molecular structure of TOI and its dimer belong to the *D*_2h_ and *D*_4h_ point group, respectively. The molecular structures of the NiNc-containing molecules were optimized at the B3LYP/D3(BJ) level using the def2-TZVP basis sets. The electronic structure calculations were performed with Turbomole.^65–67^

Various kinds of conjugated linker molecules were used to connect the TOI molecules to larger conjugated structures that can form stacked dimers. We used aromatic and antiaromatic molecular rings with two ethynyl substituents as linker molecules between the antiaromatic TOI molecules. We also constructed linked TOI molecules with butadiynyl substituted cyclooctatetraene (COT) linkers because butadiynyl is a long, stiff and linear spacer that reduces the repulsion between the antiaromatic rings. The molecular structures of the linked TOI molecules and their stacked dimers were optimized at the B3LYP level using the def2-SVP basis sets.^62^ They were optimized without symmetry constraints, except for TOI with butadiynyl linkers, which was optimized assuming the *C*_4h_ point group. We also constructed an infinite surface consisting of TOI rings connected by ethynyl substituted COT rings and its stacked dimer. The VESTA program was used for visualizing the molecular structures of the TOI-containing molecules.^68^ The Cartesian coordinates of the molecular structures are reported in the zip file of the SI.

We constructed NiNc dyads, a NiNc triad and a NiNc tetrad, where the NiNc units are connected at the C_β_–C_β_ and C_β_–C_meso_–C_β_ positions. Previous studies of one-dimensional TOI arrays, whose TOI units were fused at the C_β_–C_meso_–C_β_ positions showed that the arrays have alternating antiaromatic TOI rings and aromatic naphthalene moieties between them.^69^ We also used ethynyl moities as linkers to increase the distance between the NiNc moieties. We extended the study to molecular structures of stacked dimers of NiNc-containing molecules and to an infinite band containing connected NiNc rings.

The molecular structures of the infinite surfaces were optimized at the DFT level using the PBE functional using the pob-TZVP-rev2 basis sets, which have been optimized for periodic calculations.^70,71^ The electronic structure of the periodic systems was calculated at the CAM-B3LYP level using the optimized molecular structure and the pob-TZVP-rev2 basis sets. The calculations on the periodic structures were performed with Turbomole.^72,73^ The Brillouin-zone sampling was treated with Monkhorst–Pack *k*-point meshes,^74^ where the geometry optimization was performed at the Γ point and the electronic structure calculations were carried out with a denser 3 × 3 × 1 *k*-point grid.

The nuclear magnetic resonance (NMR) shielding constants of the TOI-containing molecules were calculated at the DFT level using the B3LYP and CAM-B3LYP functionals in combination with the def2-TZVP basis sets,^75,76^ which have been found to describe magnetic properties of large antiaromatic molecules well.^22^ The NMR shielding constants of the NiNc-containing molecules were calculated at the B3LYP level using def2-TZVP basis sets. The NMR shielding tensors were calculated with Turbomole.^65–67^

Orbital interaction diagrams were constructed using Multiwfn.^77,78^ The non-covalent interactions between the stacked dimers were studied using the interaction region indicator (IRI) method,^79^ which is implemented in the Multiwfn program. The IRI method is a generalization of the reduced density gradient approach.^80^

### 2.1 Determining the degree of aromaticity

The magnetically induced current density (MICD) susceptibilities were calculated with the GIMIC program^25,34–37^ using density matrices obtained in the NMR shielding calculations, basis-set information, and the optimized molecular structures. The strength of the MICD pathways was obtained by integrating the MICD passing through selected planes perpendicular to the molecular plane and parallel to the direction of the external magnetic field. The strength of the magnetically induced ring currents (MIRC) was also obtained by integrating the *zz* component (σ_*zz*_) of the magnetic shielding tensor along a vertical line in the center of molecular rings or inside the MIRC pathway.^81^

The harmonic oscillator model of aromaticity (HOMA)^82,83^ was used to estimate the aromatic nature of the monomers and dimers. The bond-length alternation of the C–C bonds was used to assess the aromatic character of the infinite surfaces.

The diatropic and paratropic contributions to the MICD in a given point in space were determined by following the vector field around the entire vortex using the Runge–Kutta algorithm.^84,85^ The MICD of the diatropic and paratropic vortices was stored separately and analyzed. Pictures showing the MICD pathways were made with Paraview.^86^

## 3 Results and discussion

### 3.1 Tetraoxa-isophlorin and its dimer

The HOMO–LUMO gap, the HOMA values calculated for the C–C bonds of the tetraoxa-isophlorin (TOI) rings, and the distance between them in the stacked TOI dimer are reported in Table 1. We used a reference C–C bond length of 1.39 Å in the HOMA calculations. The HOMA values calculated at the B3LYP level are in closer agreement with those calculated at the MP2 level than with the HOMA values obtained in the CAM-B3LYP and ωB97X calculations, suggesting that the aromatic nature of TOI and its dimer is qualitatively described at the B3LYP level, even though the antiaromatic character is exaggerated in the B3LYP calculations. The HOMA value of TOI calculated at the MP2 level is 0.79, whereas for the dimer it is 0.94, suggesting that the antiaromatic TOI becomes aromatic when forming the dimer. The distance between the TOI molecules of 2.93 Å is shortest at the MP2 level, which may to some extent be due to the BSSE that is larger at *ab initio* correlation levels of theory than at DFT levels. At the B3LYP level, the intermolecular distance is 3.10 Å. The TOI molecules optimized at the CAM-B3LYP and ωB97X levels are slightly bent. The intermolecular distance at these levels of theory is shorter than obtained at the B3LYP level. The HOMA calculations at the CAM-B3LYP and ωB97X levels suggest that there is no transition from antiaromatic TOI monomers to an aromatic dimer.

**Table 1:** The HOMO–LUMO gap (in eV) and the HOMA value of tetraoxa-isophlorin (TOI) and its dimer calculated at the MP2/def2-SVP level and at DFT levels using the def2-TZVP basis sets. The distance (in Å) between the two molecules of the stacked dimer is also reported

	B3LYP	CAM-B3LYP	ωB97X	MP2
**Monomer**				
Gap	1.45	3.74	5.13	5.96^a^
HOMA	0.73	0.59	0.69	0.79

**Dimer**				
Distance	3.10	3.07	2.98	2.93
Gap	1.55	3.86	5.66	4.62^a^
HOMA	0.95	0.61	0.55	0.94

a The orbital energies were obtained in the Hartree-Fock calculations.

The binding energy of the dimer was calculated at the MP2 level using the def2-SVP, def2-TZVP, and def2-QZVP basis sets. The calculated binding energies at the MP2 level using systematically increasing basis sets are 336.5, 372.8, and 368.5 kJ mol^−1^ showing that the dimer is strongly bound at the MP2 level and that the binding energies obtained with the def2-TZVP basis set are accurate. Since MP2 often overbinds, we performed single-point MP2 calculations at the spin-component-scaled MP2 (SCS-MP2)^87^ and the scaled opposite-spin (SOS-MP2)^88^ levels. The BSSE corrected binding energy is 359.2 kJ mol^−1^ at the MP2 level, 176.4 kJ mol^−1^ at the SCS-MP2 level and 85.0 kJ mol^−1^ at the SOS-MP2 level showing that the dimer is strongly bound at the MP2 levels of theory.

The isotropic ^1^H NMR shielding constants of TOI calculated at the B3LYP level are somewhat larger than those obtained using the CAM-B3LYP functional, suggesting that the paratropic ring current is stronger at the B3LYP level. Calculations of the MIRC strengths at the MP2 and B3LYP levels suggest that the antiaromatic nature of TOI is overestimated at the B3LYP level, since the MIRC strength is −64.0 nA T^−1^ at the B3LYP level, whereas the MP2 calculation yields a MIRC strength of only −27.4 nA T^−1^.^32^ The B3LYP functional often exaggerates the antiaromatic nature of large molecular rings,^32,89^ whereas the aromatic character of aromatic rings is well described at the B3LYP level.^90–93^ The MIRC strengths obtained by numerically integrating the current density passing through a plane that cuts the molecular ring agree well with the ones obtained by numerical line integration of the *zz* component of the ^1^H NMR shielding tensor (*σ*_*zz*_) along the symmetry axis in the middle of the ring (Table 2).

**Table 2:** The average isotropic ^1^H NMR shielding constant (*σ̄* in ppm) as well as the diatropic (Dia) and paratropic (Para) contributions to the MIRC strengths (*I* and *I*(*z*_0_) in nA T^−1^) of TOI and its dimer. The magnetic properties were calculated at the B3LYP and CAM-B3LYP levels using the molecular structure optimized at the B3LYP level. The diatropic and paratropic contributions to the MIRC strengths were obtained by numerical integration of the MICD passing through a plane that cuts the molecular ring(s), whereas the *I*(*z*_0_) values were obtained by numerical line integration of *σ*_*zz*_ along the symmetry axis in the center of the ring(s)

Level	*σ̄*_meso_	*σ̄*_β_	Dia	Para	*I*	*I*(*z*_0_)
**Monomer**						
B3LYP	38.57	33.93	1.91	−65.36	−63.45	−64.00
CAM-B3LYP	33.05	30.01	2.51	−37.85	−35.34	−35.76

**Dimer**						
B3LYP	23.13	23.04	66.70	−6.19	60.51	58.50
CAM-B3LYP	23.02	22.87	65.34	−6.22	59.12	59.92

**Fig. 2:**
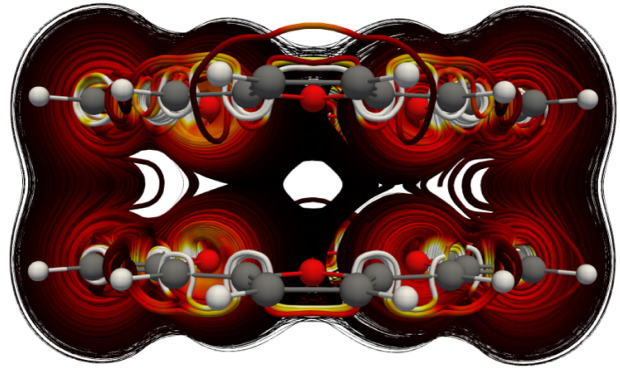
The MICD of the TOI dimer obtained with the external magnetic field applied parallel to the TOI planes.

Calculation of the MICD of the TOI dimer with the external magnetic field along the *x* axis, *i.e.*, parallel to the molecular planes shows that the dimer sustains a strong through space current of 22.4 nA T^−1^ from one TOI molecule to the other. The vertical MICD is shown in Fig. 2.

### 3.2 The aromatic bonding

The aromatic bonding can be understood from irreducible representations and energy levels of the frontier orbitals and from the transition moments between the ground state and the lowest electronic states. We use TOI and TOI_2_ as an example. The HOMO–LUMO gap vanishes for TOI when its molecular structure belongs to the *D*_4h_ point group. Jahn-Teller distortion leads to a symmetry-broken molecular structure belonging to the *D*_2h_ point group. Its HOMO–LUMO gap is 3.80 eV at the CAM-B3LYP level. The orbital energies of the HOMO and LUMO of the Jahn-Teller distorted TOI are −5.30 eV and −1.50 eV and they belong to the *B*_2g_ and *B*_2g_ irreducible representation, respectively, which means that the excitation from HOMO to LUMO is electric dipole forbidden but magnetic dipole allowed leading to the antiaromatic nature of TOI.^31^

**Fig. 3:**
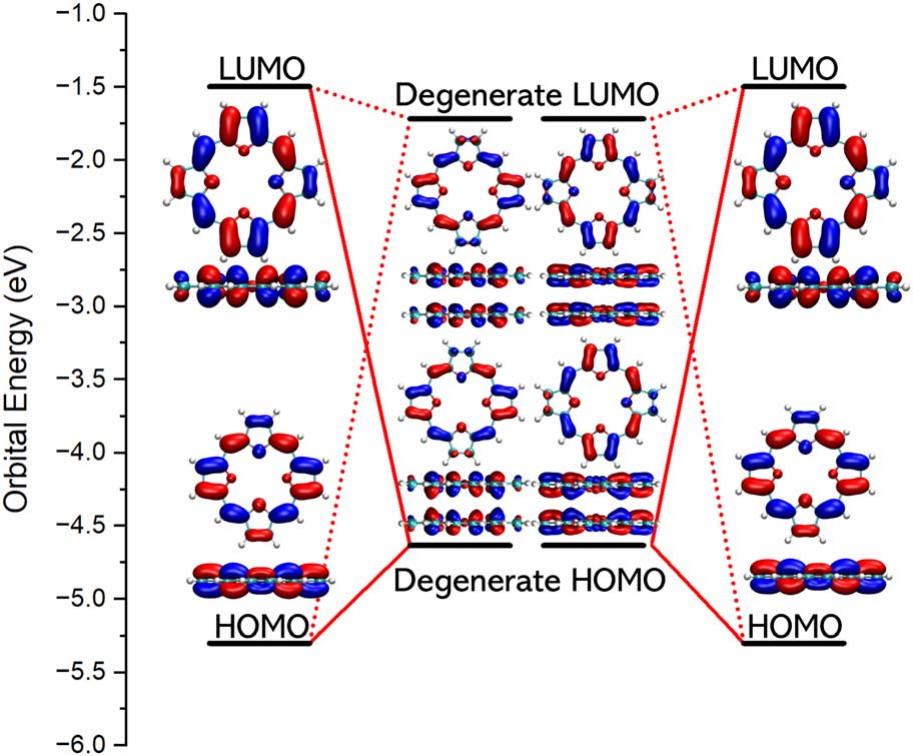
The frontier orbitals of TOI and TOI_2_.

Assuming that two TOI molecules of *D*_4h_ symmetry interact in a face-to-face fashion, the HOMO of the dimer is degenerate and filled with four electrons. It consists of the binding combination of the degenerate HOMO–LUMO pair of the non-interacting TOI molecules of *D*_4h_ symmetry. The orbital energies of the HOMO and LUMO of the stacked TOI dimer are −4.63 eV and 1.72 eV and they belong to the *E*_u_ and *E*_g_ irreducible representations, respectively. HOMO-1 belongs to the *A*_2u_ irreducible representation. The HOMO → LUMO transition is electric dipole allowed for the *z* component. The HOMO-1 → LUMO transition is electric dipole allowed in the *x*, *y* direction. The electric dipole-allowed transitions are prerequisite for the aromatic nature of the dimer.^31^ The orbital diagrams of TOI and its dimer are shown in Fig. 3.

The excitation energies of the three lowest electronic excited states of the stacked TOI dimer are 0.536 eV (*B*_2u_), 0.707 eV (*A*_1u_) and 0.794 eV (*B*_1u_). The transition from the *A*_1g_ ground state to these three states originating from the HOMO–LUMO transition is electric-dipole and magnetic-dipole forbidden. The fourth excited state (*E*_u_) at 2.001 eV is dipole allowed in the *x*, *y* direction leading to the aromaticity of the dimer. The excitation is dominated by the *E*_u_ → *B*_2g_ orbital transition. The excitation energy of the fifth excited state (*A*_2u_) is 2.111 eV, whose ground-state transition is electric-dipole allowed in the *z* direction leading to the vertical aromaticity. The *A*_2u_ state is dominated by the HOMO → LUMO transition.

### 3.3 Linker molecules

The linker molecules that connect the TOI rings consist of aromatic and antiaromatic molecular rings with linear substituents like ethynyl and butadiynyl groups as well as only the linear spacers to increase the distance between the rings. The ring-shaped linker molecules are benzene, pyrazine, and cyclooctatetraene (COT), whose molecular structure is bent and almost nonaromatic and belongs to the *D*_2d_ point group,^94,95^ The COT rings are planar in the stacked dimers.

We also constructed extended NiNc-containing molecules by connecting them at the C_β_–C_β_ and C_β_–C_meso_–C_β_ positions. The distance between the NiNc rings was extended by using ethynyl groups.

The strength of the aromatic bonding is expected to increase when the molecule contains many independent antiaromatic molecular rings because each ring contributes to the binding energy. The aim here is to design stiff two-dimensional double-layered materials with a strong interaction between the two layers due to the aromatic stabilization when changing from antiaromatic monomer rings to aromatic rings in the dimer.

### 3.4 Linked tetraoxa-isophlorin

#### 3.4.1 Benzene and pyrazine linkers

Benzene substituted with two ethynyl moieties in the para positions is chosen as an aromatic linker that connects two antiaromatic TOI rings (1). The separated diatropic and paratropic contributions to the MICD in the SI show that the TOI rings of 1 are antiaromatic and the benzene ring is aromatic. Structure optimization of the dimer (1_2_) leads to a short distance of 3.10 Å between the TOI rings. The aromatic nature of the TOI rings changes from antiaromatic to aromatic in the dimer. Since the TOI rings force the benzene rings closer to each other, the distance between the benzene rings is 3.50 Å, which is slightly shorter than the van der Waals distance of 3.9 Å for two parallel benzene rings.^96^ The benzene rings are slightly twisted with respect to the plane of the TOI rings. The short face-to-face distance between the stacked TOI moieties of 1_2_ is almost the same as for two unsubstituted TOI molecules. The longer intermolecular distance between the aromatic linkers shows that there is only an ordinary van der Waals interaction between the stacked aromatic rings.^1,2^ The molecular structures of 1 and 1_2_ are shown in the SI.

The ^1^H NMR shielding constants of the β and meso protons of TOI show that 1 is antiaromatic. The ^1^H NMR shielding constants of the benzene linker suggest that it is aromatic but less aromatic than a single benzene molecule. The antiaromatic TOI rings of 1 are aromatic in the stacked 1_2_ dimer. The HOMO–LUMO gap of 2.61 eV for the dimer is smaller than the one for 1 of 3.21 eV. In 1_2_, the ^1^H NMR shielding constants are in the typical range for aromatic porphyrinoids. The HOMA value increases from 0.72 in 1 to 0.92 in 1_2_ showing that stacking reduces the bond-length alternation, which is a geometric evidence for the change from antiaromatic to aromatic nature. The HOMO–LUMO gap, the HOMA values and the ^1^H NMR shielding constants are given in Table 3.

**Table 3:** The HOMO–LUMO gap (in eV), the HOMA values and the isotropic ^1^H NMR constants (*σ* in ppm) are reported for 1, 1_2_, 2 and 2_2_

Molecule	Gap	HOMA	*σ*_meso_	*σ*_*β*_	*σ*_linker_
1	3.21	0.72	33.22	29.80	25.43
1_2_	2.61	0.92	23.07	22.68	23.35
2	3.39	0.71	33.12	29.80	27.84
2_2_	2.66	0.92	22.96	22.59	24.86

Separating the diatropic and paratropic contributions to the MICD shows that 1 is antiaromatic because it is dominated by paratropic contributions, whereas 1_2_ is aromatic with dominating diatropic MICD contributions. The strength of the MIRC of two stacked TOI rings in 1_2_ is 55.84 nA T^−1^, which is about twice the MIRC strength of free-base porphyrin.^97^

Pyrazine substituted with two ethynyl groups connected to the nitrogen atoms in the para positions is used as an antiaromatic linker between the TOI rings in 2. The ^1^H NMR shielding constants suggest that the TOI and pyrazine rings in the twisted 2 are antiaromatic. The stacked TOI rings of 2_2_ are parallel, whereas the linker molecules are rotated out of the TOI plane as the benzene rings in 1_2_. The molecular structures of 2 and 2_2_ are shown in the SI.

The ^1^H NMR shielding constants of the stacked TOI rings of 2_2_ are typical for aromatic porphyrinoids. The strength of the MIRC of the stacked TOI rings is 56.95 nA T^−1^ showing that the TOI dimer is strongly aromatic, while the MIRC strength of the TOI ring in the monomer is −37.61 nA/T. The pyrazine linker is antiaromatic with a MIRC strength of −10.13 nA T^−1^ in 2. They are also antiaromatic in 2_2_ with a MIRC strength of −5.13 nA T^−1^ suggesting that there is no aromatic bonding between the pyrazine rings.

The HOMA values increase, which supports the notion that the TOI rings are aromatic in the dimer. The stacking distance of the TOI rings is 3.10 Å, whereas the stacking distance of the rotated pyrazine rings is 3.50 Å showing that there is a repulsion between them as between the benzene rings in 1_2_. The diatropic and paratropic contributions to the MICD of 2 and 2_2_ are similar to those of 1 and 1_2_, respectively. The HOMO–LUMO gap, HOMA values and ^1^H NMR shielding constants of 2 and 2_2_ are reported in Table 3.

#### 3.4.2 Cyclooctatetraen linker

Cyclooctatetraene (COT) has a non-planar molecular structure belonging to the *D*_2d_ point group. It is nonaromatic sustaining a weak paratropic MIRC of −2.82 nA T^−1^.^98^ Molecule 3 is constructed by connecting two TOI rings to the two ethynyl substituents in the para positions of the COT ring. The COT ring in 3 is nonaromatic and bent, whereas the TOI rings are planar and antiaromatic as seen from the ^1^H NMR shielding constants in Table 4.

**Table 4:** The HOMO–LUMO gap (in eV), the HOMA values, and the ^1^H NMR shielding constants (in ppm) are reported for the TOI-containing molecules with COT linkers

Molecule	Gap	HOMA	*σ*_meso_	*σ*_β_	*σ*_linker_
3	3.34	0.72	33.18	29.80	26.67
3_2_	2.17	0.91	24.12	23.28	23.49
4_2_	1.98	0.91	22.54	21.31	22.24
Periodic 4_2_	1.86	0.93	—	—	—
5_2_	1.67	0.89	25.22	25.55	24.97
6_2_	1.81	0.94	23.01	21.43	22.98

**Fig. 4:**
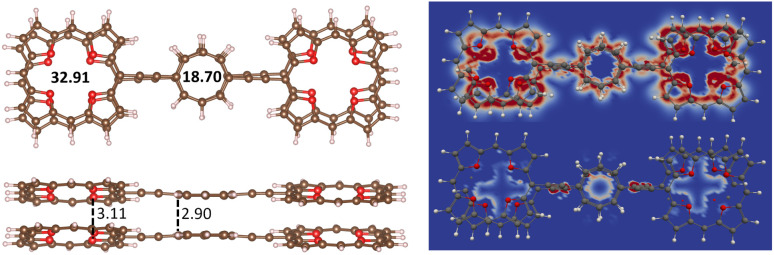
The molecular structures of 3_2_ are shown with the distances between the oxygen atoms of the TOI rings and distance between the carbon atoms of the COT linkers. The MIRC strengths of the TOI and COT rings were obtained by using Ampère-Maxwells law. The right picture shows the diatropic (upper) and paratropic (lower) contributions to the MICD of 3_2_.

The stacked 3_2_ dimer consists of two almost planar 3 molecules, where the TOI rings are slightly twisted. The HOMO–LUMO gap of 3_2_ of 2.17 eV is about 1 eV smaller than for 3. The binding energy of 3_2_ calculated at the MP2 level is 569.1 kJ mol^−1^,which is slightly smaller than twice the binding energy of the TOI dimer of 359.2 kJ mol^−1^ because 3 is bent. The intermolecular distance between the oxygen atoms of the TOI rings of 3_2_ is 3.11 Å and the COT distance is 2.90 Å. The molecular structure of 3_2_ is shown in Fig. 4 and the molecular structure of 3 is shown in the SI. The HOMA value of the TOI ring is larger in 3_2_ than for 3 suggesting its aromatic character changes from antiaromatic 3 to aromatic 3_2_. The ^1^H NMR shielding constants in Table 4 are consistent with this interpretation. The MIRC strength of the stacked TOI rings of 3_2_ is 32.91 nA T^−1^ and the stacked COT rings sustain a MIRC of 18.70 nA T^−1^. Calculating the diatropic and paratropic contributions to the MICD shows that 3_2_ is dominated by diatropic MICD. The paratropic contribution appears in the inner part of the rings as for benzene. The diatropic and paratropic contributions to the MICD are shown in Fig. 4 The HOMO–LUMO gap, the HOMA values and ^1^H NMR shielding constants of 3 and 3_2_ are reported in Table 4.

**Fig. 5:**
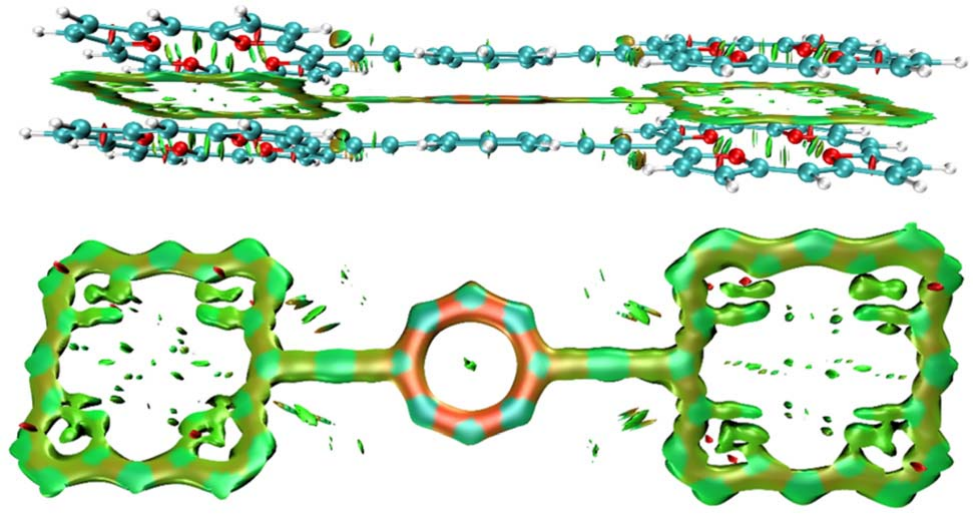
The chemical bond between the TOI and COT rings of 3_2_ is illustrated by the IRI plot.

The non-covalent interaction between the stacked 3_2_ dimer is illustrated with the interaction region indicator (IRI) method. The IRI plot in Fig. 5 shows that the stacking interaction is delocalized over the whole molecule. Thus, all three antiaromatic rings contribute to the binding of the dimer. Delocalized double bonds are formed between each pair of stacked rings.

**Fig. 6:**
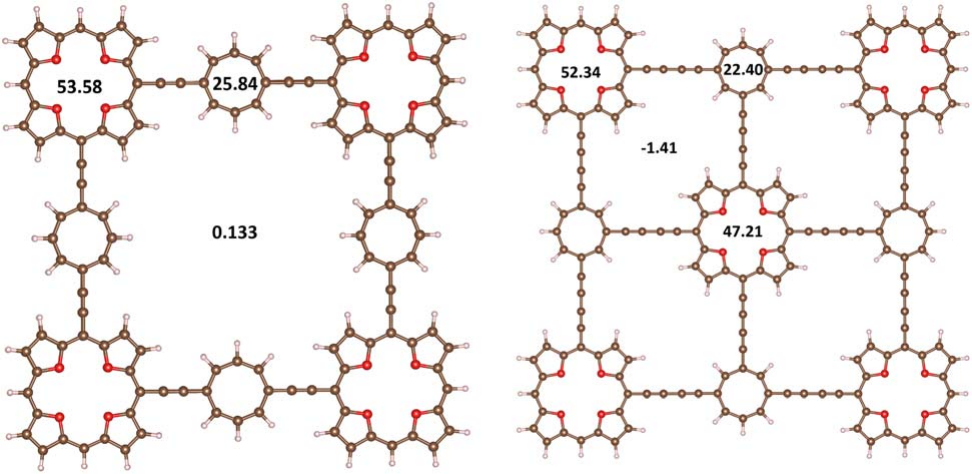
The molecular structures of 4_2_ (left) and 6_2_ (right) are shown. The MIRC strengths of the TOI and COT rings were obtained using Ampère–Maxwells law.^81^

Rigid planar molecules were constructed by connecting four (4) or five (5) TOI rings with four COT linkers. The molecules consisting of many antiaromatic rings form strongly bound stacked dimers because each antiaromatic ring contributes to the binding energy of the dimer, when they change from antiaromatic to aromatic nature. The TOI and the COT rings of 4_2_ are planar and parallel with a short distance of 3.10 Å between the monomers. The molecular structures of 4_2_ and 5_2_ are shown in Fig. 6 and in the SI, respectively. The HOMA value of the TOI rings of 4_2_ is 0.91, which is the same as for 3_2_. In the molecular structure of 5_2_, the TOI rings in the middle are distorted because there is not enough space for them, as shown in the SI. The HOMA value of 5_2_ of 0.89 is therefore somewhat smaller than for 4_2_. We replaced the ethynyl bridges with butadiynyl moieties to increase the distance between the rings.

Molecule 6_2_ shown in Fig. 6 is an aromatic dimer with five TOI rings connected by four butadiynyl-substituted COT rings. All rings sustain strong local diatropic MIRC, whereas almost no net MIRC flows around the whole molecule. The TOI pair of rings in the corners sustains a local MIRC of 52.34 nA T^−1^ and the middle one sustains a MIRC of 47.21 nA T^−1^. The COT dimers sustain a diatropic MIRC of 22.40 nA T^−1^. Thus, all nine antiaromatic rings of 6 become aromatic when forming the 6_2_ dimer.

The HOMO–LUMO gap of 4_2_ is 1.98 eV and due to steric effects, the gap of 1.67 eV is slightly smaller for 5_2_. The ^1^H NMR shielding constants of 4_2_ suggest that it is aromatic. The ^1^H NMR shielding constants of 5_2_ are larger than for 4_2_ due to the congested TOI in the middle of the molecule. The HOMO–LUMO gaps, the HOMA values and the ^1^H NMR shielding constants are given in Table 4.

The optimized molecular structure of the periodic 4_2_ has a smaller bond-length alternation than 4_2_, since the HOMA value of the TOI rings is 0.93 for the periodic structure, whereas it is 0.91 in 4_2_.

**Fig. 7:**
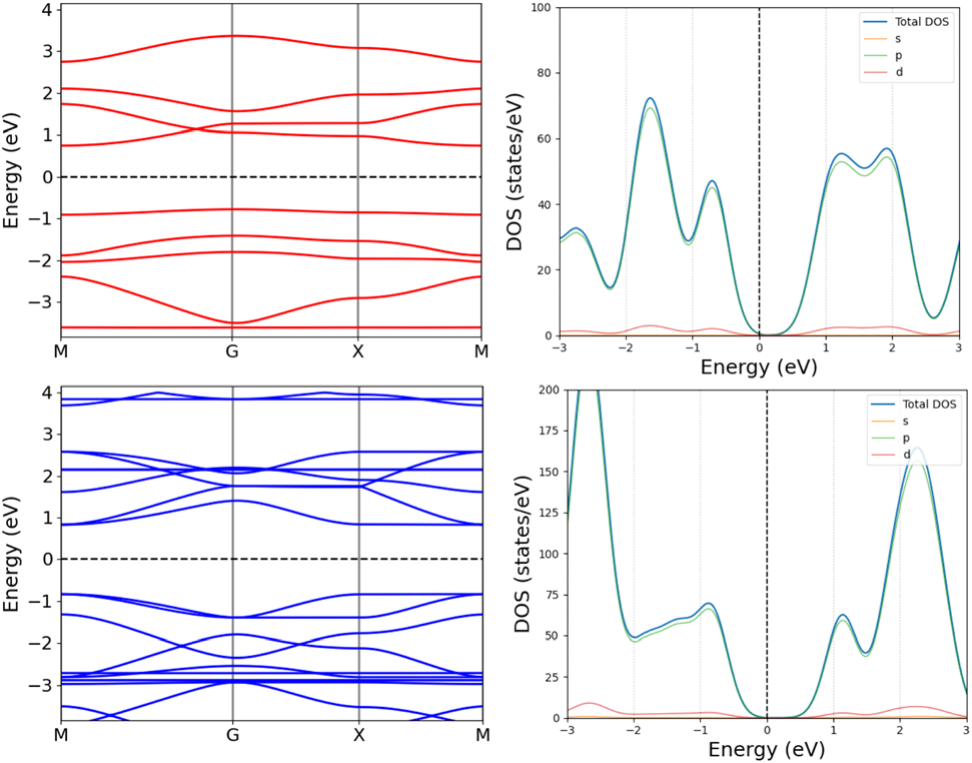
The band structures (left) and density of states (right) of the periodic 4 (top) and the periodic 4_2_ dimer (bottom).

The one layer periodic 4 has an indirect band gap of 1.56 eV, whereas the double-layer periodic 4_2_ has a direct band gap of 1.86 eV, which is close to the band gap of 1.98 eV for 4_2_. The changes in the band structure shown in Fig. 7 can be attributed to the coupling between the two layers in the periodic dimer.

**Fig. 8:**
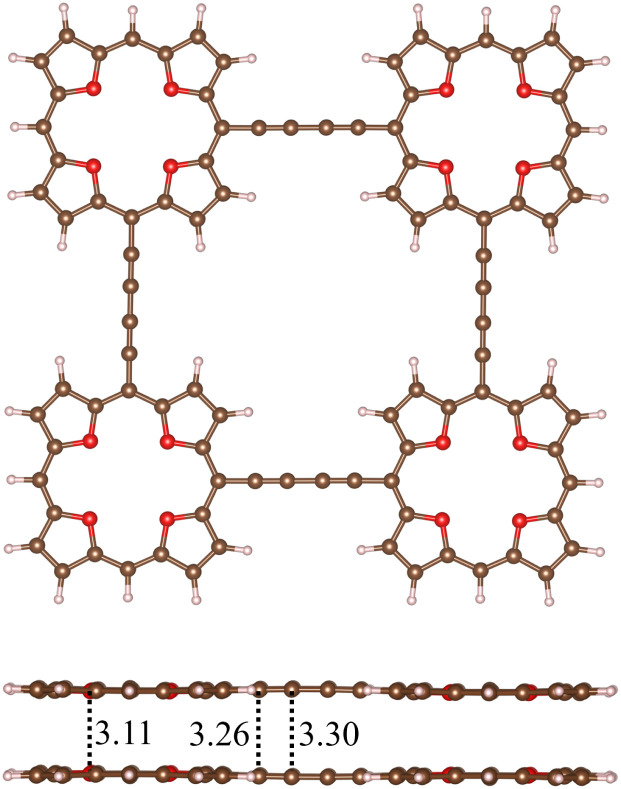
The molecular structures of the quadratic butadiyne-linked TOI (7) and its dimer (7_2_) are shown.

A quadratic molecule consisting of four TOI molecules connected with butadiyne linkers (7) is shown in Fig. 8. Butadiyne linked porphyrinoids may be easier to synthesize than when using molecular rings in the linkers.^99^ Molecule 7 is planar and forms a planar stacked dimer (7_2_) with a distance of 3.11 Å between the planes at the TOI molecules. There is a weak repulsion between the butadiyne linkers leading to a stacking distance of 3.30 Å between them.

### 3.5 Linked Ni(ii)-norcorrole

**Fig. 9:**
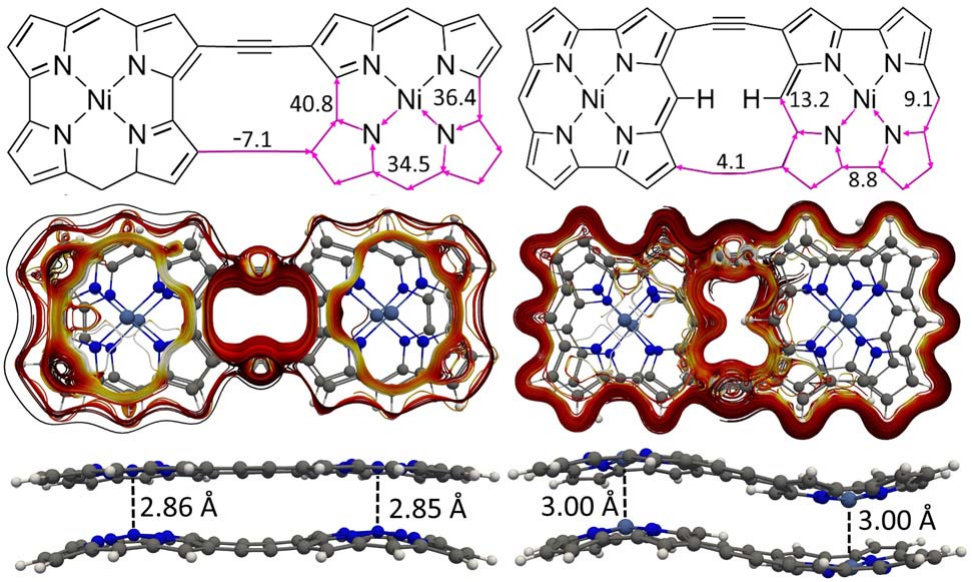
The molecular structures, MIRC strengths, MICD pathways of 10_2_ (left) and (11)_2_ (right) are shown.

**Fig. 10:**
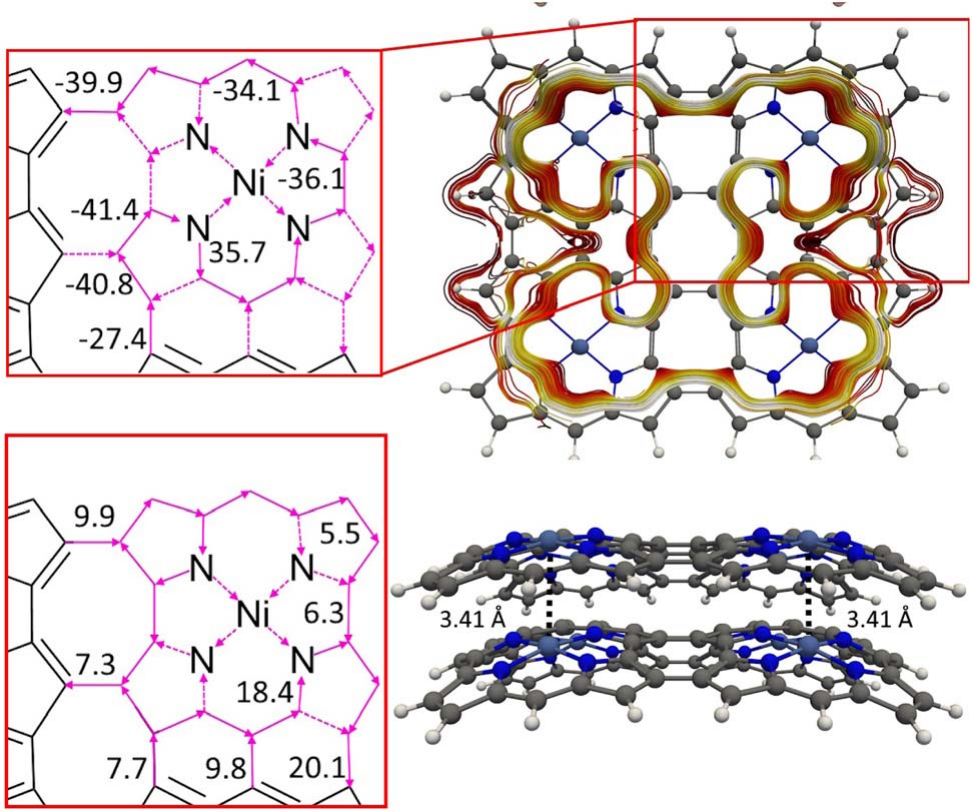
The MICD pathways and MIRC strengths of the NiNc-based tetrad (13) (above) and its dimer (13)_2_ (below).

We constructed NiNc dyads by connecting two NiNc molecules at two C_β_ positions (8) and by connecting them at two C_β_ positions and at the meso position between them (9). The distance between the NiNc rings in the dyads was also increased by ethynyl linkers at the C_*β*_ positions (10 and 11). The molecular structures of 10 and 11 are shown in Fig. 9 and in the SI. A linear triad was constructed by connecting three NiNc moieties in the C_β_ positions (12). The molecular structures of 8, 9, and 12 are shown the SI. A quadratic tetrad (13) was obtained by fusing the NiNc moieties in the C_β_ and C_meso_ positions. We also investigated the aromatic character of stacked dimers of the dyads, the triad and the tetrad. The molecular structures of (13) and (13)_2_ are shown in Fig. 10. They are minima obtained from different initial geometries. More pictures of the molecular structures and MIRC strengths of the NiNC molecules are shown in the SI.

We calculated the MIRC pathways of 8, 9 and their stacked dimers. The obtained MIRC strengths and the HOMA values of these molecules are reported in Table 5. The C_β_–C_meso_–C_β_ connection of the NiNc rings leads to a strong interaction between NiNc units that changes their aromatic character. 9 sustains a diatropic MIRC of 16.4 nA T^−1^ around the central naphthalene-pyrrole moiety and a MIRC of 5.7 nA T^−1^ along the perimeter. A similar MIRC pattern was previously obtained for arrays of porphyrins and TOIs connected at the C_β_–C_β_ and C_β_–C_meso_–C_β_ positions.^69^ The stacked 9_2_ dimer is aromatic. It has the same kind of naphthalene–pyrrole conjugated moiety that sustains a local MIRC. The Ni–Ni distance of 3.74 Å indicates that it has no aromatic bonding between the NiNc moieties.

**Table 5:** The MIRC strengths (*I* in nA T^−1^), HOMA values, and Ni–Ni distances (in Å) of the NiNc structures calculated at the DFT level using the B3LYP functional. The periodic structure was optimized using the PBE functional and its electronic structure was calculated using the CAM-B3LYP functional

Structure	Monomer		Dimer		
	*I* (nA T^−1^)	HOMA	*I* (nA T^−1^)	HOMA	Ni–Ni (Å)
8	−95.4	0.42	34.9	0.44	2.862
9	5.7	0.52	11.4	0.48	3.744
10	−73.8	0.45	36.4	0.45	2.856
11	−52.8	0.47	9.1	0.43	3.000
12	−88.6	0.39	36.0	0.46	2.910
13	−36.1	0.40	6.3	0.31	3.414
8_∞_^a^	—	0.47	—	0.50	2.693
10_∞_^a^	—	0.61	—	0.58	2.759

a 1D periodic system (infinite band).

Connecting the NiNc rings at the C_β_–C_β_ positions in 8 leads to a weak interaction between the NiNc rings implying that it sustains a strong local paratropic MIRC of −95.4 nA T^−1^ in each NiNc ring. There is also a weak paratropic current of −6.2 nA T^−1^ along the perimeter of the 8 dyad implying that it is globally antiaromatic, which agrees with previous experimental and computational studies of the dyad.^52^ The stacked 8_2_ dimer is aromatic sustaining strong diatropic MIRC of 34.9 nA T^−1^. It sustains a paratropic MIRC of −9.9 nA T^−1^ around the eight-membered ring between the NiNc rings. It has a short Ni–Ni distance of 2.86 Å.

The NiNc moieties of the 12 triad *i.e.*, an array of three NiNc units, sustain a strong local paratropic MIRC of −88.6 nA T^−1^ in the NiNc rings and a weak paratropic MIRC of −4.1 nA T^−1^ along the perimeter of the triad. The (12)_2_ dimer is aromatic with a Ni–Ni distance of 2.91 Å, which is somewhat longer than the Ni–Ni distance of 2.86 Å for the dimer of the corresponding dyad. The 12 monomers of the dimer are equidistant but slightly bent. The NiNc rings of (12)_2_ sustain a diatropic MIRC of 36.0 nA T^−1^, whereas the strength of the global paratropic MIRC is −6.6 nA T^−1^.

The change from antiaromatic to aromatic character upon dimerization is also reflected in the HOMA values. For 8 and 12, the HOMA values are 0.42 and 0.39, respectively, which are smaller than for 8_2_ and (12)_2_, whose HOMA values are 0.44 and 0.46, respectively.

**Fig. 11:**
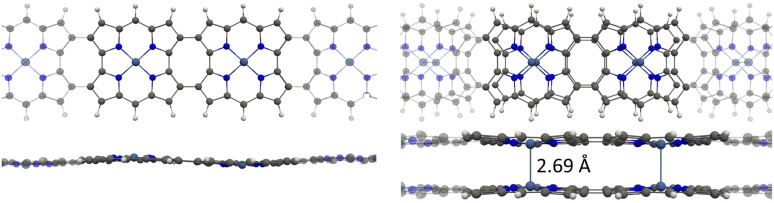
Top and side views of the 1D periodic structures 8_∞_ and (8_∞_)_2_ (right). The corresponding cell parameters is 16.70 Å for both. Atoms outside the unit cell are faded out.

**Fig. 12:**
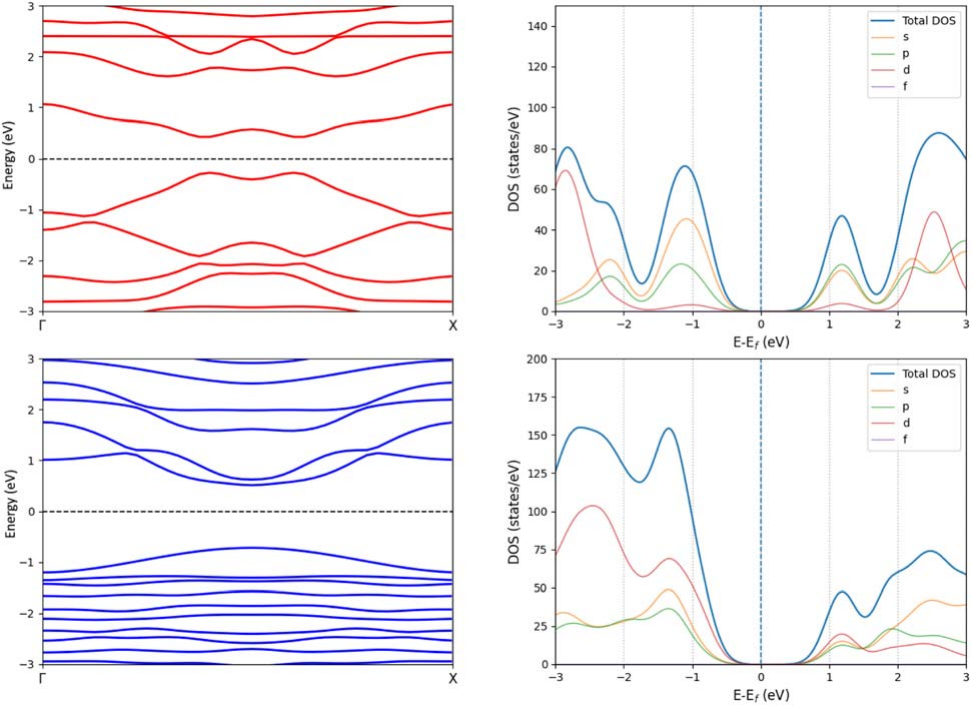
The band structures (left) and density of states plots (right) of the periodic 8_∞_ (top) and (8_∞_)_2_ (bottom).

We constructed the one-dimensional (1D) periodic structures 8_∞_, (8_∞_)_2_, 10_∞_, and (10_∞_)_2_, whose NiNc rings are connected through two C_β_–C_β_ bonds, either directly as in 8_∞_ and (8_∞_)_2_ (Fig. 11), or *via* ethynyl bridges as in 10_∞_ and (10_∞_)_2_. The HOMA value of 8_∞_ is 0.44, whereas the infinite (8_∞_)_2_ dimer has a HOMA value of 0.56, indicating a higher degree of aromaticity for the periodic dimer structure than for the periodic monomer, which is consistent with what we obtained for 8_∞_ and 12. 8_∞_ and (8_∞_)_2_ have a direct band gap, as seen in the band structure and density of states plots in Fig. 12. In 8_∞_, the valence and conduction bands are mainly formed by s and p orbitals, whereas the contribution from the d orbitals of the Ni atoms is significant in the dimer.

Ethynyl linkers were introduced to extend the C_β_–C_β_ connection bridge. The ethynyl groups prevent the formation of strong global MICD pathways in the 10 and 11 structures shown in the SI. 11 sustains local MIRCs in the NiNc rings rather than around the entire molecule as in the directly connected 9 dyad. A weak paratropic MIRC of −2.7 nA T^−1^ passes across the ethynyl bridges that connect the two NiNc units. The NiNc rings of 10 sustain a local paratropic MIRC of −73.8 nA/T. The strong antiaromaticity of the NiNc rings leads to the formation of the stacked aromatic 10_2_ and (11)_2_ dimers shown in Fig. 9. The ethynyl linkers of 8_2_ does not affect the aromatic bonding of the dimer, since the Ni–Ni distances of 2.86 Å are the same in 8_2_ and 10_2_. The ethynyl linkers of 9_2_ also promotes the aromatic bonding in (11)_2_, although the interaction is weaker than in 10_2_, as indicated by the longer Ni–Ni distance of 3.00 Å.

**Fig. 13:**
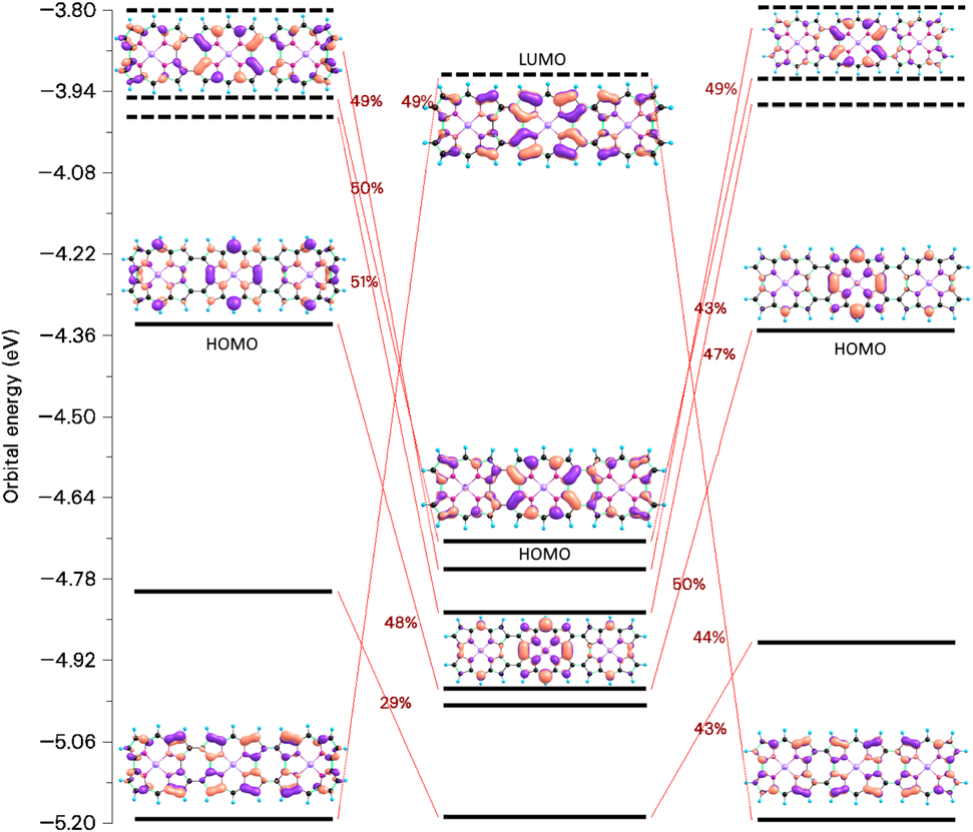
The molecular orbitals and the orbital interaction diagram of (12)_2_ and the mixing of the monomer orbitals when forming the dimer. The molecular structures of the monomers in the dimer differ because the Multiwfn program^77,78^ used for making the orbital diagram requires that the monomers have the same molecular structure as they have in the dimer.

The chemical bond between the monomers of the stacked (12)_2_ dimer can be analyzed by constructing the orbital interaction diagram shown in Fig. 13. The two monomers in (12)_2_ have almost the same molecular structures and the order and energy of the orbitals are almost the same. The stacked (12)_2_ dimer has six bonding orbitals. Four orbitals binds the two monomers in 8_2_ and (NiNc)_2_ has two orbitals forming a chemical bond between the stacked NiNc rings,^20^ which suggests that for NiNc-(ββ-NiNc)_*n*_)_2_ (*n* = 0, 1, 2, …) there is one double bond per stacked NiNc pair. The bonding orbitals shown in Fig. 13 are delocalized over the whole dimer and their shape is reminiscent of the monomer orbitals from which they originate. Since the HOMA values of (8_∞_)_2_, 8_2_, and (12)_2_ are larger than for the monomer, we conclude that there is one double bond per stacked NiNc pair also in the infinite bands.

For 12 and 8, the HOMA value increases when forming the dimer, while for the other NiNc-based compounds, the HOMA value of the stacked dimer is smaller than for the monomer. Despite the strong antiaromaticity of the NiNc rings of (11)_2_, the number of binding orbitals is according to the interaction diagram shown in the SI less than three due to the distortion of the molecular structure, which is caused by the repulsion between hydrogen atoms in the middle of the molecule.

**Fig. 14:**
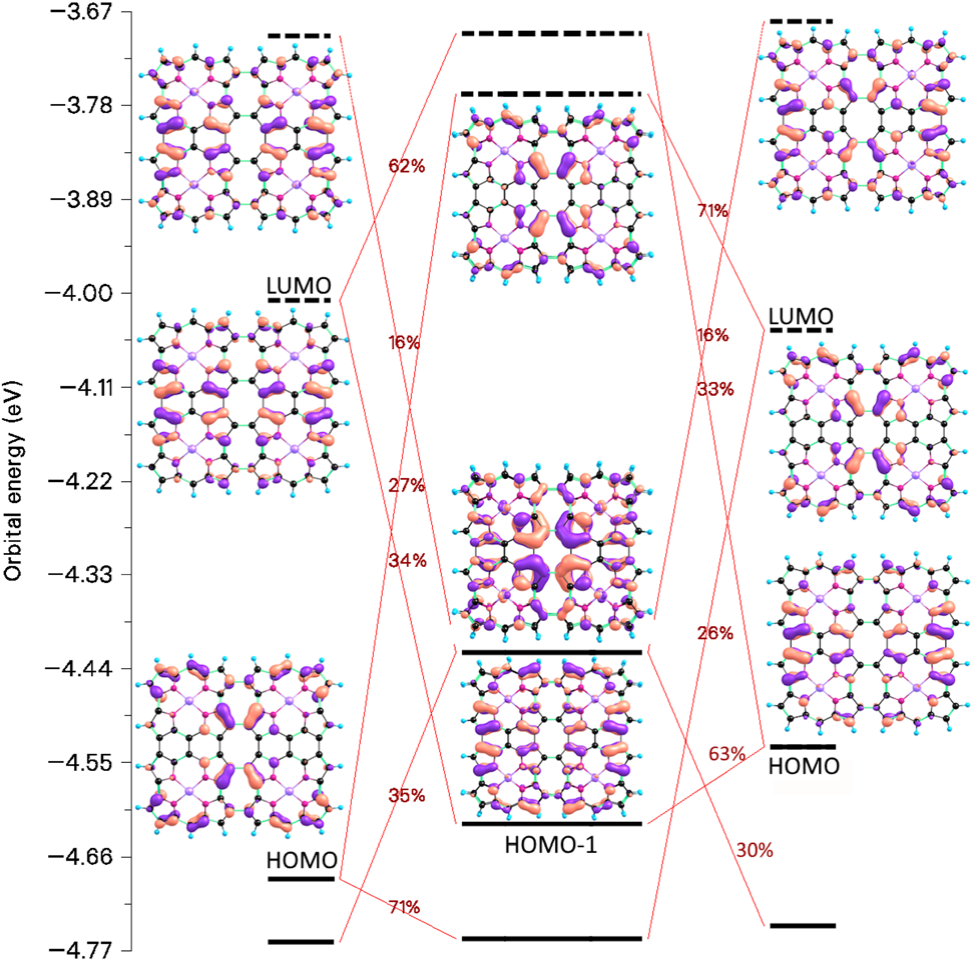
The molecular orbitals and the orbital interaction diagram of (13)_2_ and its monomers. The molecular structures of the monomers in the dimer differ because the Multiwfn program^77,78^ used for making the orbital diagram requires that the monomers have the same molecular structure as they have in the dimer.

From the orbital interaction diagrams of (13)_2_ in Fig. 14, it can be seen that the orbital energies and the order of frontier orbitals of the monomers in (13)_2_ differ significantly due to the different structures of the monomers. Despite the long Ni–Ni distance of 3.41 Å in (13)_2_, there is double bond between its monomers. The aromatic (13)_2_ is formed by aromatic bonding from strongly antiaromatic 13 and its bonding orbitals are delocalized over the entire dimer.

## 4 Conclusions

We have studied the aromatic properties of tetraoxa-isophlorin (TOI) and its stacked dimer at different levels of theory. MP2 and B3LYP calculations show that the aromatic nature changes from antiaromatic monomers to an aromatic stacked dimer. The binding energy of the TOI dimer is 359.2 kJ mol^−1^ at the MP2 level. Calculations at the B3LYP level exaggerates the antiaromatic character of TOI, whereas the paratropic MIRC is usually weaker when using functionals with larger amount of HF exchange. Since molecular structure optimization using the CAM-B3LYP and ωB97X functionals leads to deformed TOI rings in the dimer, we optimized the molecular structures at the B3LYP level and performed calculations of the MICD at the B3LYP and CAM-B3LYP levels.

The stacking interaction between antiaromatic molecules leads to the formation of dimers that sustain a diatropic MIRC. Molecules consisting of many antiaromatic rings form dimers that are strongly bound because each pair of antiaromatic rings contributes to the binding. The IRI method shows that the non-covalent interaction between the stacked dimers is delocalized around each ring. For molecules with many antiaromatic rings, all rings contribute to the binding of the dimer since delocalized double bonds are formed between each pair of stacked rings. However, the conjugation between molecular rings can prevent the formation of the aromatic bonding because it changes the aromatic character of the connected rings. The conjugation and strong coupling between the antiaromatic rings can be reduced by introducing linkers that are connected *via* formal single bonds to the antiaromatic rings. Then, the molecule consists of independent antiaromatic rings that bind *via* aromatic bonding forming a stacked dimer.

Two TOI molecules connected with linkers consisting of benzene or pyrazine with two ethynyl groups in the *para* positions have antiaromatic TOI rings. The benzene ring of the linker is aromatic and the pyrazine ring is antiaromatic. The short stacking distance and the change in the aromatic nature of the TOI rings show that there is a strong interaction between them, whereas the longer distance between the two benzene rings and between the two pyrazine rings show that there is only an ordinary van der Waals interaction between the aromatic benzene rings and the antiaromatic pyrazine rings in the dimer.

The antiaromatic cyclooctatetraene (COT) with two ethynyl groups in the *para* positions is an antiaromatic linker that becomes aromatic in the stacked dimer. The molecule consisting of two TOI rings with a COT linker has three antiaromatic rings that become aromatic in the stacked dimer. The distance between the stacked tetraoxa-isophlorin molecules is 3.11 Å and 2.90 Å between the stacked COT rings suggesting that larger molecular double-decker structures can be constructed by alternating COT and tetraoxaphlorin rings. The distance between the rings can be extended by using ethynyl or butadiynyl spacers. The strength of the chemical bond between the monomers of the stacked dimer increases with the number of antiaromatic rings in the monomer. Calculations on a quadratic ring-shaped molecular structure consisting of four TOI rings connected with four ethynyl-substituted COT rings showed that its dimer consists of locally aromatic stacked TOI dimers and aromatic stacked COT dimers. The molecule is globally non-aromatic. Connecting four TOI rings analogously with four butadiynyl linkers also lead to a quadratic dimer with locally aromatic stacked TOI rings. Since calculations on the molecule with a TOI ring in the middle showed that there is not enough space for it, we used butadiynyl spacers to increase the distance between the rings. MICD calculations showed that the stacked rings are also in this case aromatic and that the stacked dimer is globally non-aromatic. Calculations with periodic boundary conditions on a surface consisting of TOI rings with ethynyl substituted COT rings as linker molecules showed that the monomer consists of antiaromatic rings. The monomer surface has an indirect band gap, whereas the corresponding dimer is aromatic with a direct band gap.

We also propose double-decker molecules based on antiaromatic Ni(ii)-norcorrole (NiNc). The length of the conjugation pathway was extended by directly connecting the NiNc moieties or by using ethynyl spacers between the NiNc rings. Calculations show that double-decker molecules consisting of C_β_–C_β_ fused NiNc moieties are aromatic, whereas the monomers are antiaromatic. Even though the stacked dimers are not completely planar, they are bound with a very short distance of <3 Å between the monomers.

The interaction between the NiNc rings is stronger when fusing the NiNc rings through two C_β_ positions and one meso position, which changes the overall aromatic character of the monomer making the aromatic dimer formation less favorable. The strongest aromatic bonding of NiNc rings was obtained for one-dimensional bands whose NiNc rings are directly connected by two C_β_–C_β_ bonds. Then, the stacked dimers have two bonding orbitals per NiNc ring. The C_β_–C_meso_–C_β_ fusion of the NiNc rings in the two-dimensional NiNc tetramer leads to one double bond instead of four double bonds between the monomers. Introducing ethynyl linkers at the C_β_ positions reduces the conjugation between the NiNc rings, which strengthens the aromatic bonding of the dimer. Calculations with periodic boundary conditions on a linear band of NiNc rings show that the monomer and the stacked dimer have a direct band gap.

Our study shows that antiaromatic molecular rings form aromatic stacked dimers that are bound by four electrons of each pair of rings. A stronger binding of the dimer is obtained by designing molecules with many independent antiaromatic rings. The calculations on periodic structures show that these chemical bonds also keep infinite double-decker surfaces together with a distance of less than 3 Å between the monomer planes.

## Author contributions

QW studied the isophlorin-containing molecules and RTN the Ni(ii)-norcorrole-containing ones. They contributed equally to this work. They wrote the first version of the manuscript. DS proposed the project, supervised the calculations, and wrote the final version of the manuscript together with all authors.

## Conflicts of interest

There are no conflicts to declare.

## Supplementary Material

SC-OLF-D5SC10179D-s001

SC-OLF-D5SC10179D-s002

## Data Availability

The data supporting this article have been included as part of the supplementary information (SI). Density functional theory calculations were performed with Turbomole version 7.9. The Turbomole webpage is https://www.turbomole.org/. GIMIC, version 2.0 can be freely downloaded from https://github.com/qmcurrents/gimic and https://zenodo.org/record/8180435. ParaView and VESTA are free software that can be downloaded from https://www.paraview.org/and https://jp-minerals.org/vesta/, respectively.
