# Supplementary material for: Change of the aromatic nature through face-to-face stacking
Source: Chem Sci. 2026 Mar 27;17(19):9768–79. doi: 10.1039/d5sc10179d (PMC13054589; doi:10.1039/d5sc10179d)
Supplement: SC-017-D5SC10179D-s002 [file SC-017-D5SC10179D-s002.pdf]

Supplementary Information (SI) for Chemical Science.
This journal is © The Royal Society of Chemistry 2026

Journal Name

ARTICLE TYPE

Cite this: DOI: 00.0000/xxxxxxxxxx

ELECTRONIC SUPPLEMENTARY INFORMTION (ESI)

Change aromatic through face-to-face stacking†

Received Date
Accepted Date

DOI: 00.0000/xxxxxxxxxx

S1 Linked Tetraoxa-isophlorins and their dimers

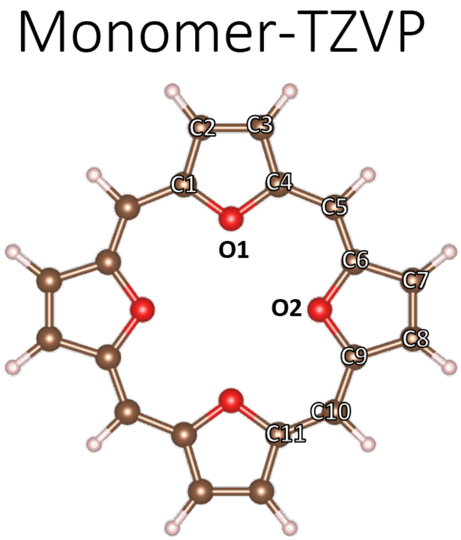

O1-C1-C2-C3-C4-O1:
B3lyp: 1.380-1.441-1.348-1.441-1.380
Cam-b3lyp: 1.372-1.446-1.335-1.446-1.372
Wb97x: 1.372-1.453-1.334-1.453-1.372
MP2: 1.379-1.436-1.356-1.436-1.379

O2-C6-C7-C8-C9-O2:
B3lyp: 1.368-1.375-1.413-1.375-1.368
Cam-b3lyp: 1.358-1.364-1.413-1.364-1.358
Wb97x: 1.358-1.363-1.418-1.363-1.358
MP2: 1.365-1.386-1.404-1.386-1.365

C4-C5-C6:
B3lyp: 1.350-1.425
Cam-b3lyp: 1.338-1.430
Wb97x: 1.336-1.436
MP2: 1.358-1.422

C9-C10-C11:
B3lyp: 1.425-1.350
Cam-b3lyp: 1.430-1.338
Wb97x: 1.436-1.336
MP2: 1.422-1.358

Figure S1 Selected bond lengths of tetraoxa-isophlorin.

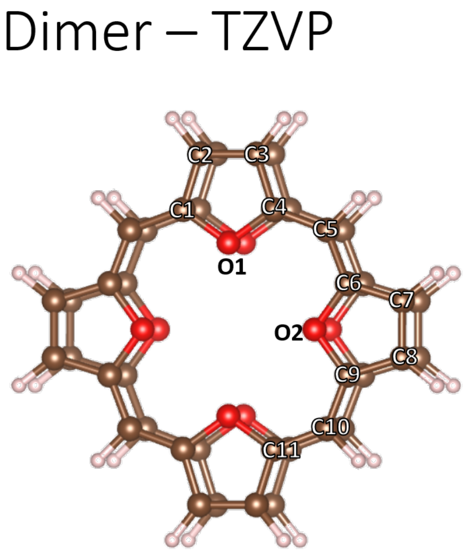

O1-C1-C2-C3-C4-O1-(layerO-C_alpha-C_beta):
B3lyp: 1.372-1.405-1.379-1.405-1.372-(3.166-3.193-3.251)
Cam-b3lyp: 1.370-1.443-1.336-1.443-1.370-(3.456-3.490-3.535)
Wb97x: 1.358-1.364-1.417-1.364-1.358-(3.244-3.296-3.358)
MP2: 1.373-1.406-1.382-1.406-1.373-(2.934-2.995-3.115)

O2-C6-C7-C8-C9-O2(layerO-C_alpha-C_beta):
B3lyp: 1.372-1.405-1.379-1.405-1.372-(3.166-3.193-3.251)
Cam-b3lyp: 1.358-1.365-1.411-1.365-1.358-(3.456-3.490-3.535)
Wb97x: 1.371-1.450-1.334-1.450-1.371-(3.243-3.296-3.359)
MP2: 1.373-1.406-1.382-1.406-1.373-(2.934-2.995-3.115)

C4-C5-C6:
B3lyp: 1.385-1.385
Cam-b3lyp: 1.339-1.427
Wb97x: 1.433-1.337
MP2: 1.389-1.389

C9-C10-C11:
B3lyp: 1.385-1.385
Cam-b3lyp: 1.427-1.339
Wb97x: 1.337-1.433
MP2: 1.389-1.389

Figure S2 Selected bond lengths of the dimer of tetraoxa-isophlorin.

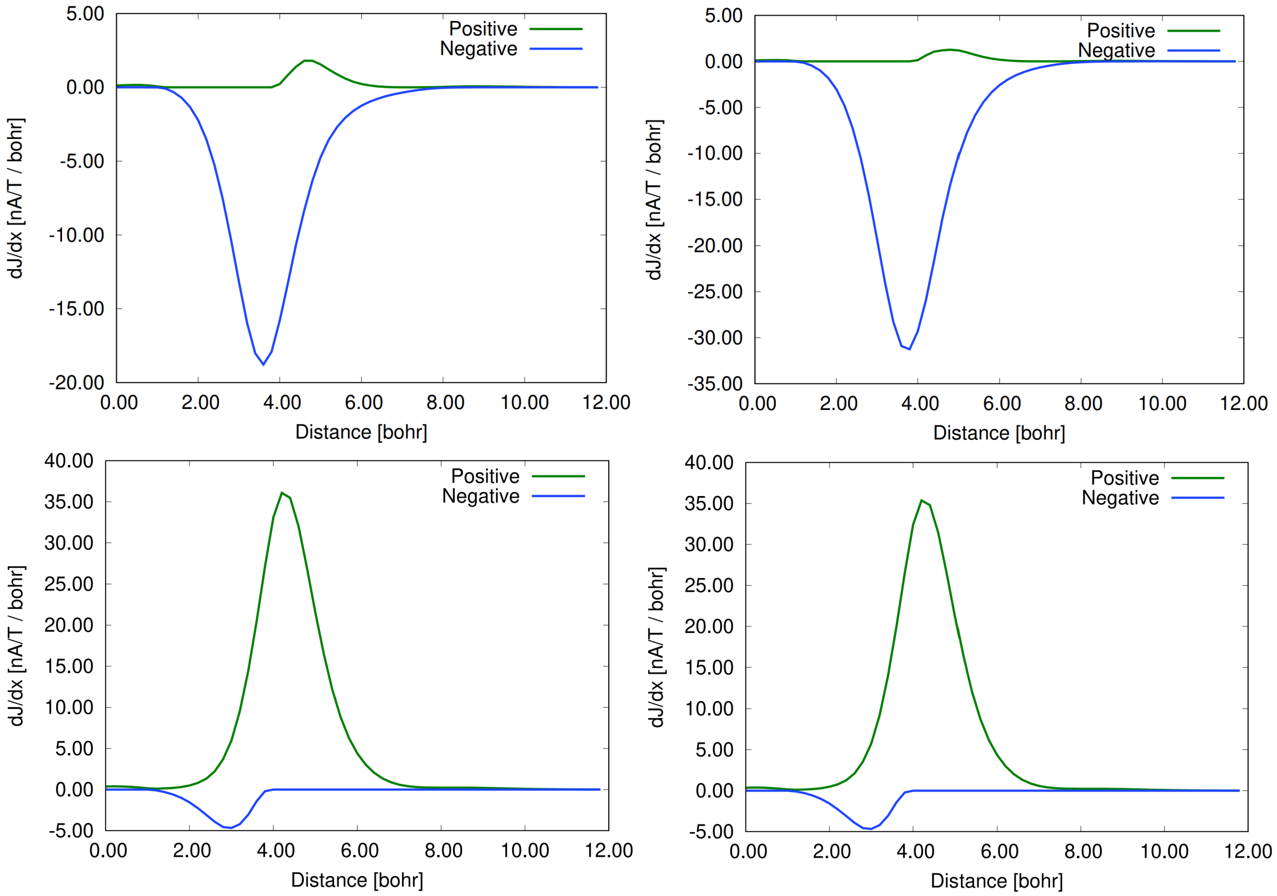

Figure S3 The ring-current profiles of tetraoxa-isophlorin (first row) and its dimer (second row) passing through a plane that is perpendicular to the molecular plane, beginning in the molecular centre and crossing the middle of C_{α}-Cmeso bond. The magnetically induced current densities are calculated at the CAM-B3LYP (left) and B3LYP (right) levels. The magnetic field is perpendicular to the molecular ring.

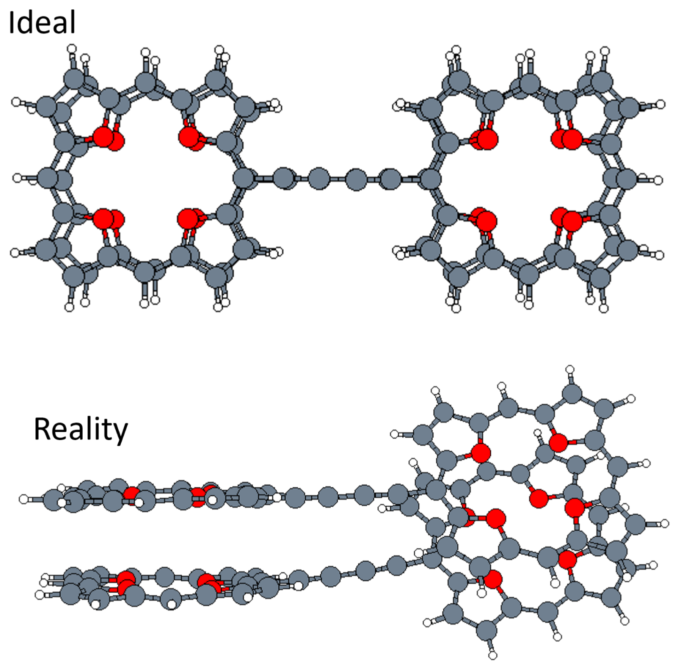

Figure S4 The ideal molecular structure of the tetraoxa-isophlorin dimer with a butadiynyl linker is compared to the optimized molecular structure.

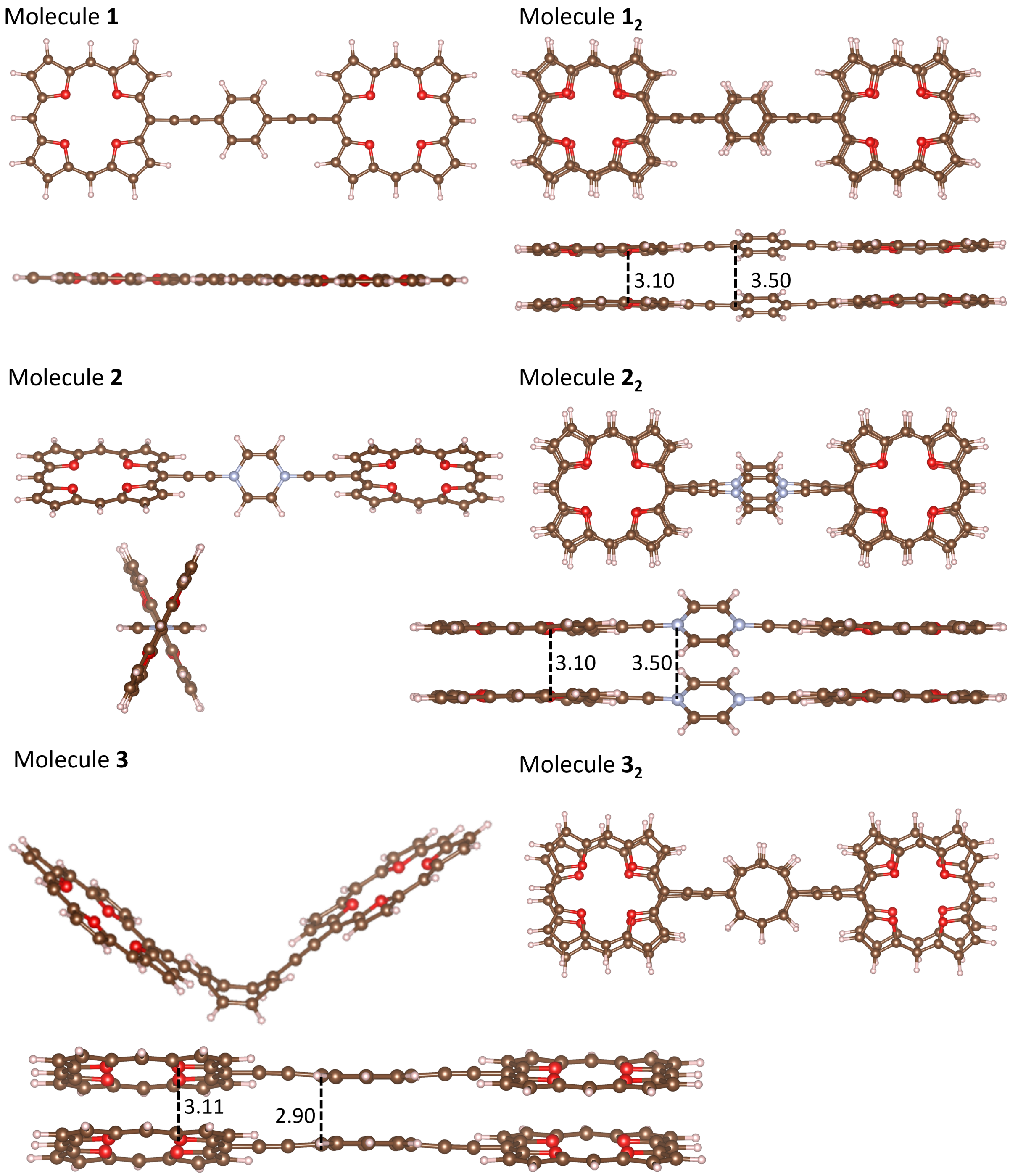

Figure S5 The side and top views of **1**, **2**, **3**, and their dimers.

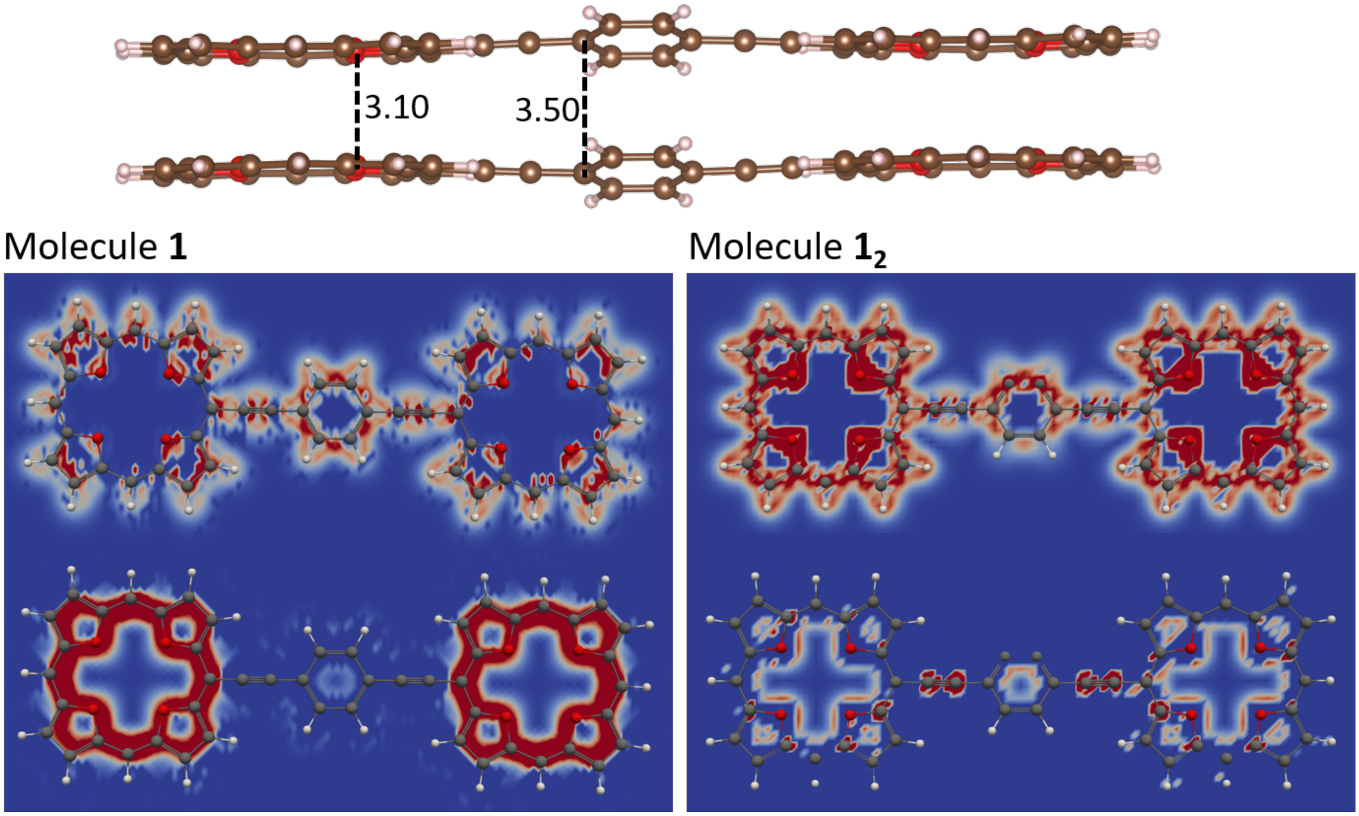

Figure S6 The molecular structure of $\mathbf{1}_2$ and the distances between the oxygen atoms of the tetraoxa-isophlorin rings and between the carbon atoms of the benzene rings are shown in the upper picture. The lower pictures show the diatropic (above) and paratropic (below) contributions to the current density of **1** (left) and $\mathbf{1}_2$ (right). The current-density strength is indicated with the color, where dark red means strong, light red means weaker, and dark blue means no current density.

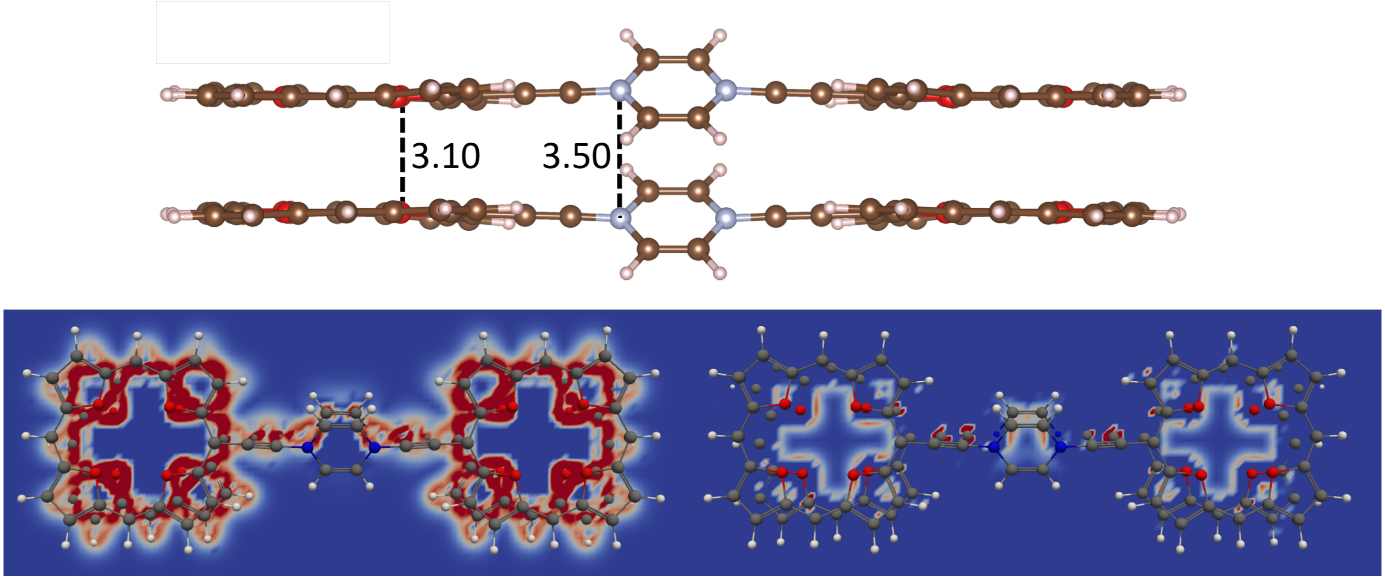

Figure S7 The molecular structure and the distances between the stacked oxygen and nitrogen atoms in $\mathbf{2}_2$ are shown. The diatropic (left) and paratropic (right) contributions to the MICD of $\mathbf{2}_2$ are also shown.

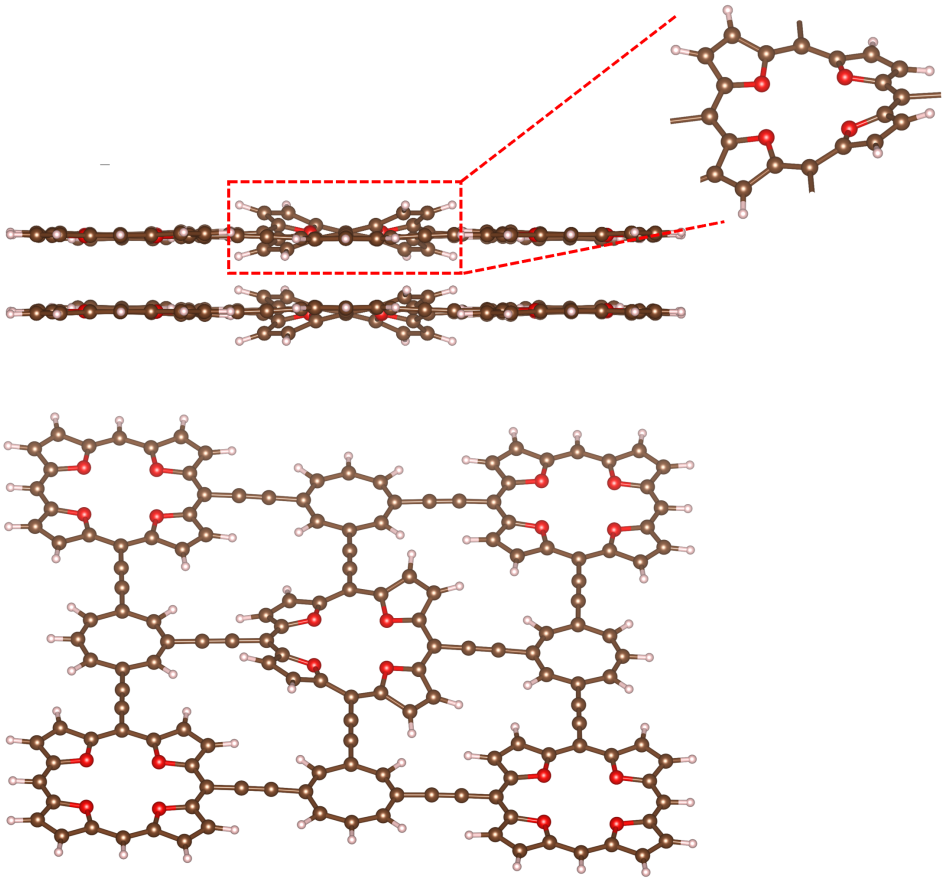

Figure S8 The side view of $\mathbf{5}_2$ and a magnified view of its deformed central region due to steric inteactions (upper). The top view of molecular structure of $\mathbf{5_2}$ is shown below it.

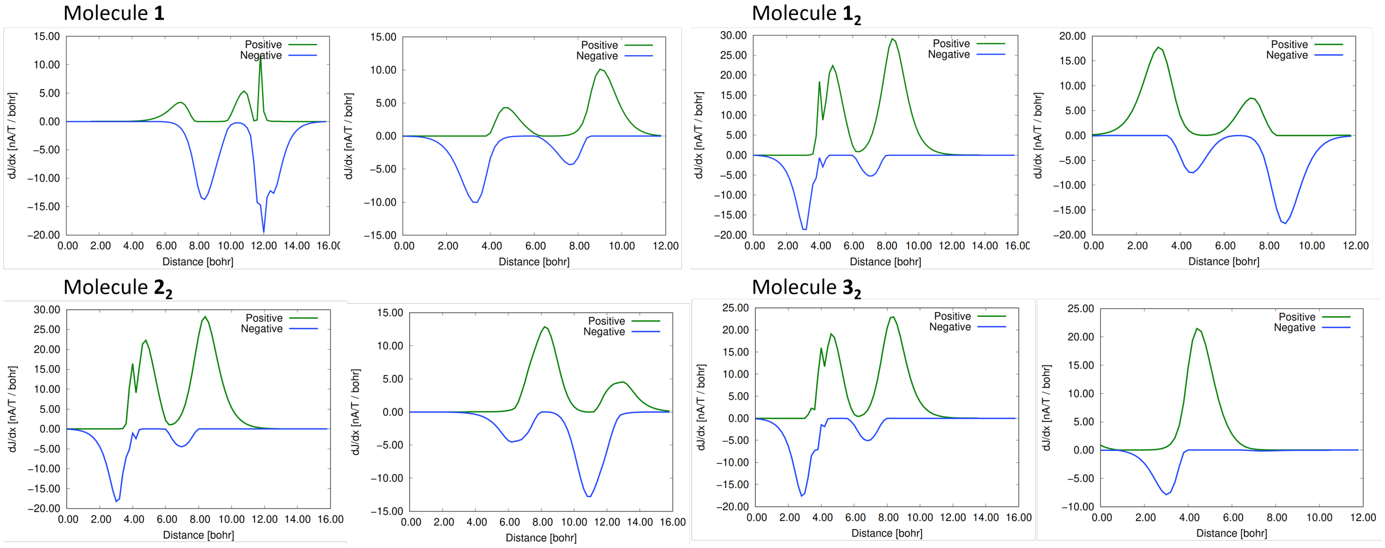

Figure S9 The ring-current of profiles of $\mathbf{1}, \mathbf{1_2}, \mathbf{2_2}, \mathbf{3_2}$.

S2 Linked Ni(II)-norcorrole

The effect of substituents on the MIRC strength of Ni(II)-norcorrole (NiNc) was investigated by stepwise substitution of pentafluorophenyl (C_6F_5) groups, which have been previously used in the synthesis of NiNc dimers. (References are given in the main article). We constructed NiNc derivatives with an increasing number of C_6F_5 substituents and studied the aromatic nature of them. Calculations of the MICD shows that the studied NiNc derivatives are strongly antiaromatic. The MIRC strength of NiNc is -63.6 nA/T. For NiNc with two C_6F_5 groups in the meso positions (NiNc-2m-C_6F_5), the MIRC strength is -67.9 nA/T and for NiNc with two C_6F_5 groups in the β positions and two C_6F_5 groups in the meso positions (NiNc-2m2$f\beta$-C_6F_5), the MIRC strength is -61.8 nA/T. NiNc with two C_6F_5 in the meso positions and four C_6F_5 in the β positions (NiNc-2m4β-C_6F_5) sustains the weakest MIRC of -54.5 nA/T among the studied NiNc derivatives. Pictures of the MIRC are shown in the ESI†. Since the substituted NiNc are strongly antiaromatic and the MIRC strength is largely independent of the number of electron-withdrawing substituents, NiNc derivatives are expected to be a suitable building block for designing planar connected antiaromatic systems.

Table S1 Magnetically induced current strengths (I, in nA/T) and HOMA indices for Ni(II)-norcorrole (NiNc) derivatives with varying numbers of C_6F_5 substituents. NiNc-2m-C_6F_5 denotes NiNc with two C_6F_5 in the meso positions. NiNc-2m2β-C_6F_5 denotes NiNc with two C_6F_5 in the β positions and two C_6F_5 in the meso positions. NiNc-2m4β-C_6F_5 denotes NiNc with four C_6F_5 in the β positions and two C_6F_5 in the meso positions.

	NiNc	NiNc-2m-C_6F_5	NiNc-2m2β-C_6F_5	NiNc-2m4β-C_6F_5
I (nA/T)	-63.6	-67.9	-61.8	-54.5
HOMA	0.4271	0.4271	0.4270	0.3857

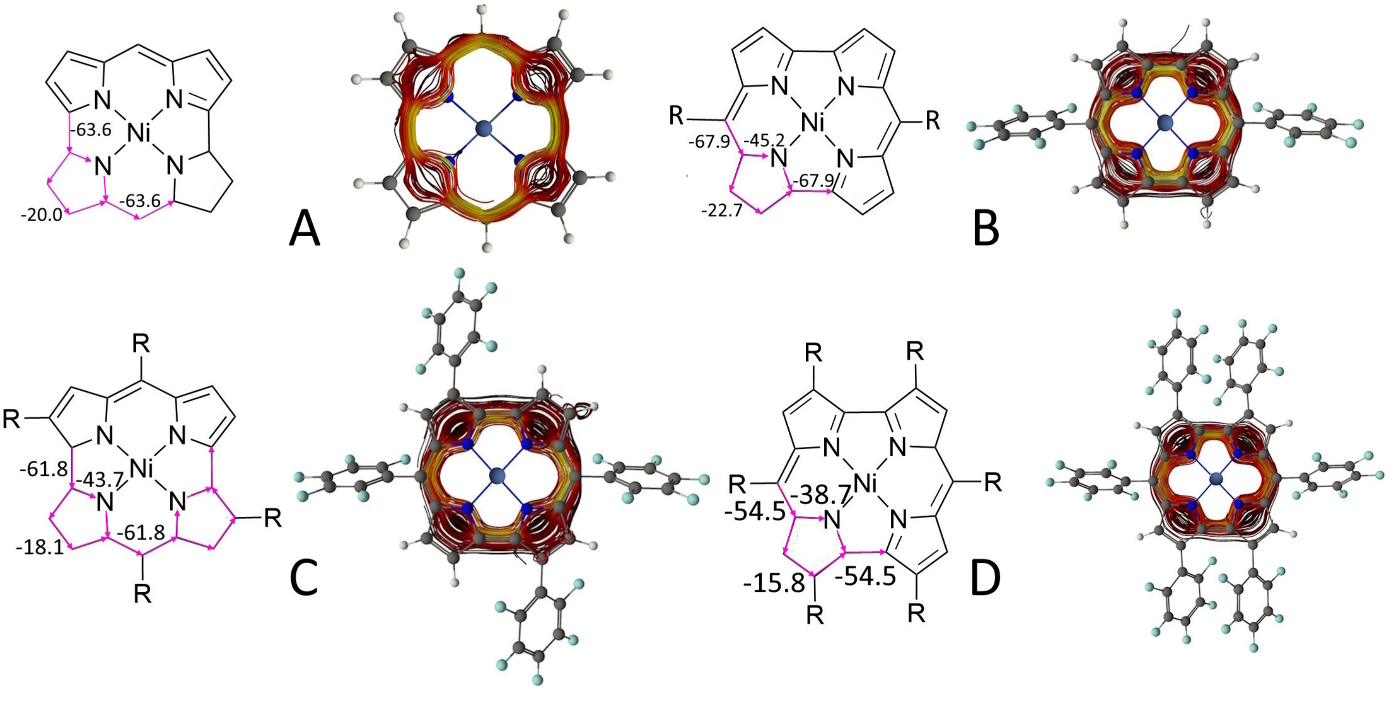

Figure S10 Magnetically induced ring current pathways in NiNc derivatives NiNc (A), NiNc-2m-C_6F_5 (B), NiNc-2m2β-C_6F_5 (C), and NiNc-2m4β-C_6F_5 (D).

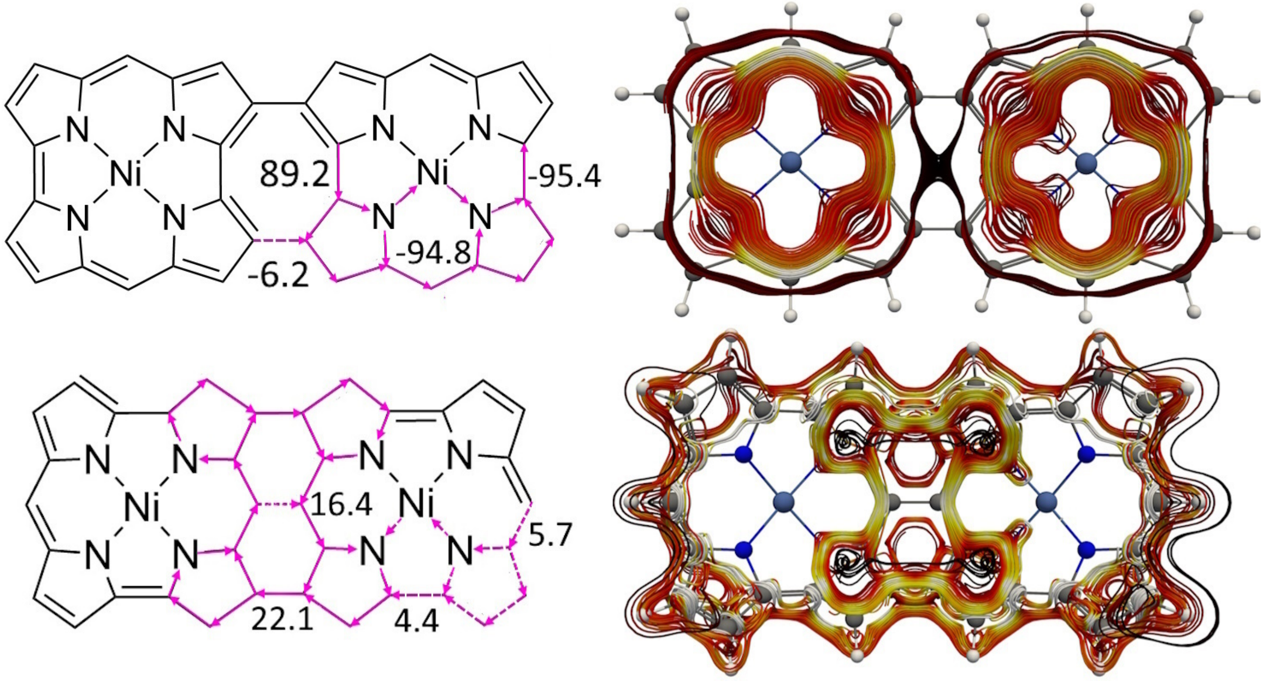

Figure S11 Current density pathways and ring-current strengths of conjugated NiNc-based structures. Molecule **8** (A) is linked in the C_β positions and molecule **9** (B) is also connected in the meso positions.

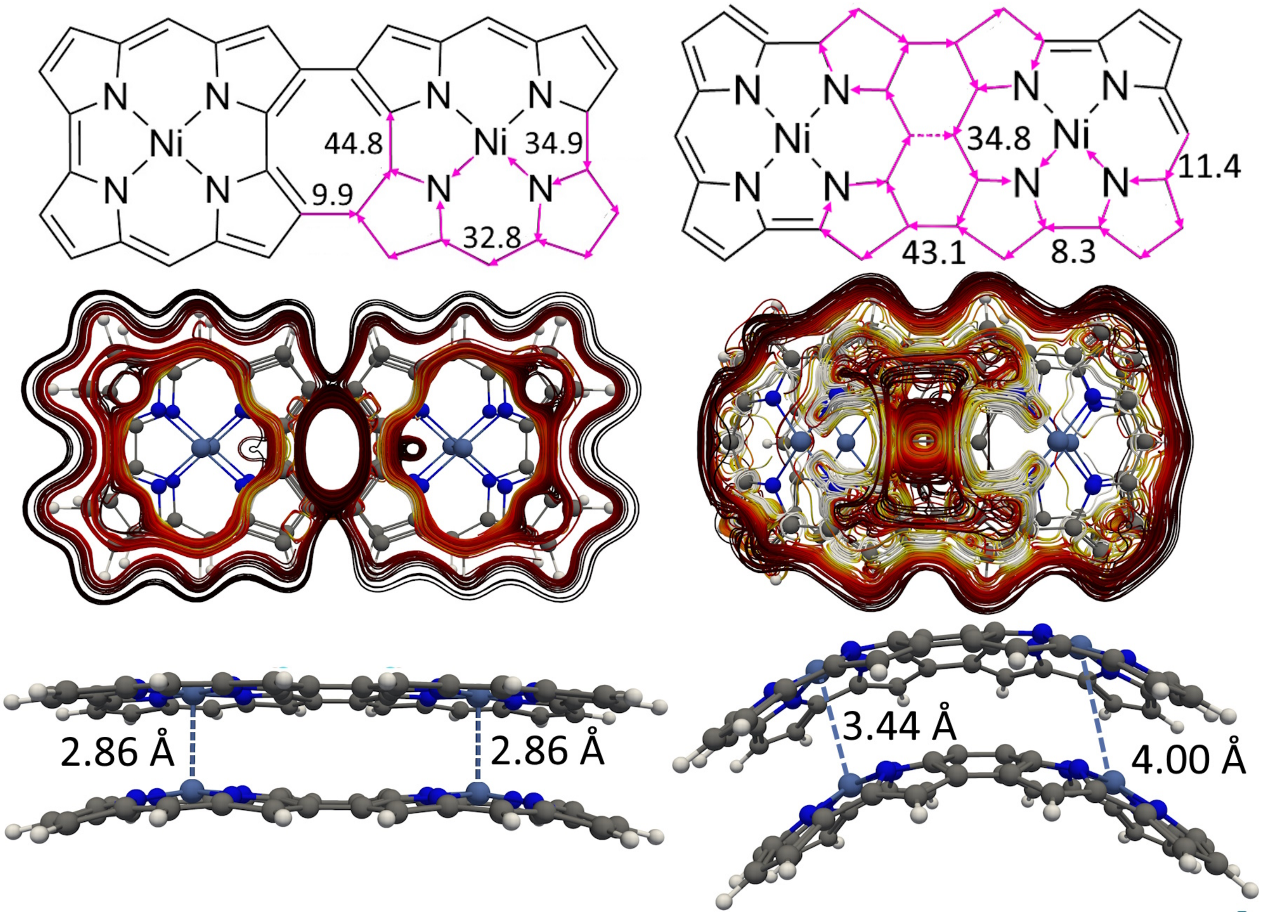

Figure S12 Current density pathways and ring-current strengths of the dimers of the conjugated NiNc-based dyads $\mathbf{8}_2$ (left column) and $\mathbf{9}_2$ (right column).

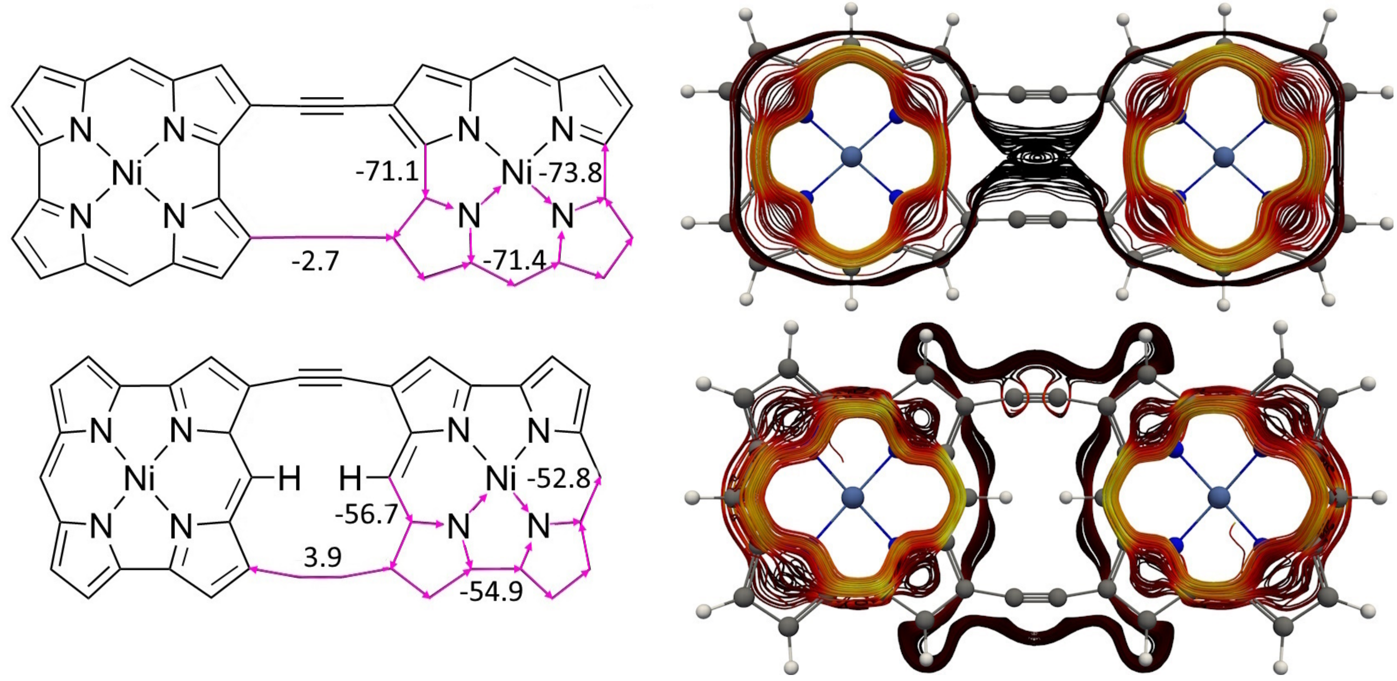

Figure S13 The current density pathways and ring-current strengths of **10** (first row) and **11** (second row).

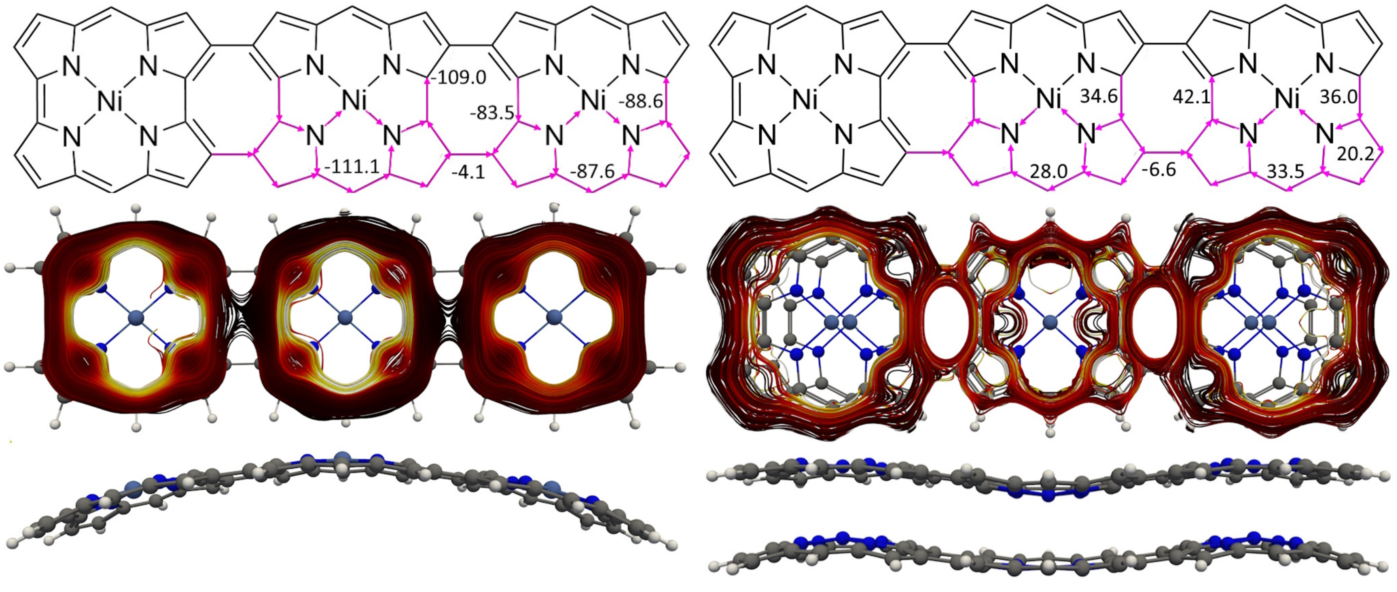

Figure S14 Current density pathways and ring-current strengths of **12** (left column) and its dimer $\mathbf{12}_2$ (right column).

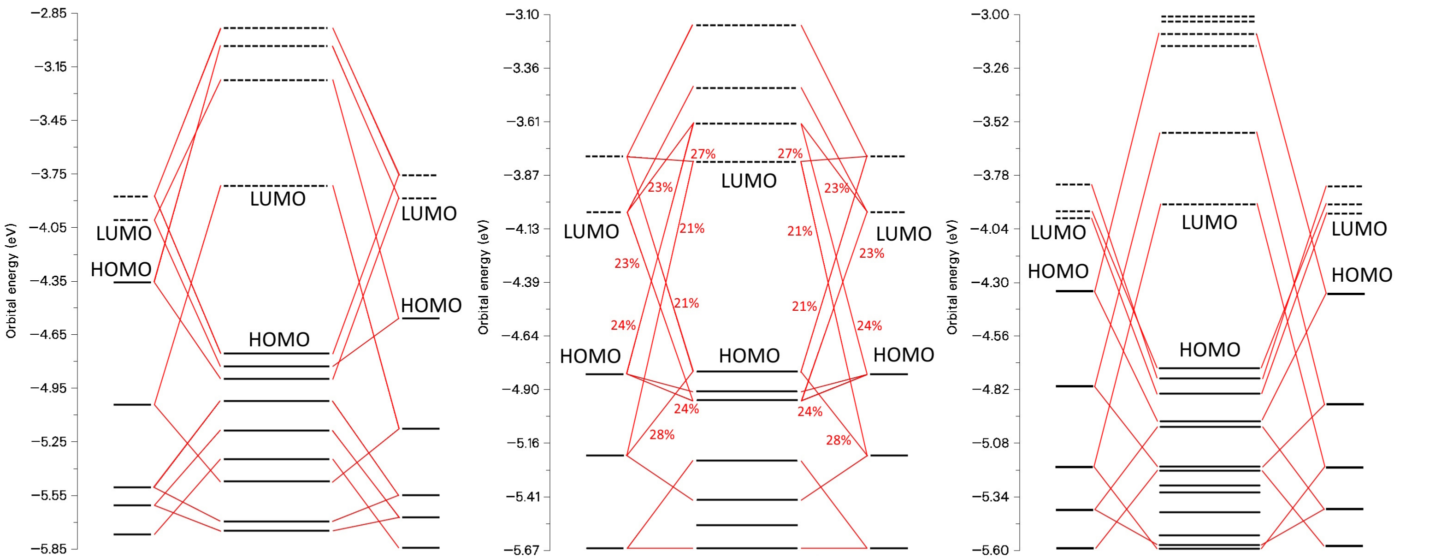

Figure S15 The orbital interaction diagrams of $\mathbf{8}_2$, $\mathbf{11}_2$ and $\mathbf{12}_2$.
